# Prognostic MRI features to predict postresection survivals for very early to intermediate stage hepatocellular carcinoma

**DOI:** 10.1007/s00330-023-10279-x

**Published:** 2023-10-23

**Authors:** Hanyu Jiang, Yun Qin, Hong Wei, Tianying Zheng, Ting Yang, Yuanan Wu, Chengyu Ding, Victoria Chernyak, Maxime Ronot, Kathryn J. Fowler, Weixia Chen, Mustafa R. Bashir, Bin Song

**Affiliations:** 1 Department of Radiology, West China Hospital, Sichuan University, Chengdu, 610041 Sichuan China; 2 Department of Technology, JD.Com, Inc, Beijing, China; 3 Department of Technology, ShuKun (BeiJing) Technology Co., Ltd, Beijing, China; 4 Department of Radiology, Memorial Sloan Kettering Cancer Center, New York City, NY USA; 5 Université Paris Cité, UMR 1149, CRI, Paris & Service de Radiologie, Hôpital Beaujon, APHP.Nord, Clichy, France; 6 Department of Radiology, University of California San Diego, San Diego, CA USA; 7 Department of Radiology, Center for Advanced Magnetic Resonance in Medicine, and Division of Gastroenterology, Department of Medicine, Duke University Medical Center, Durham, NC 27710 USA; 8 Department of Radiology, Sanya People’s Hospital, Sanya, 572000 Hainan China

**Keywords:** Carcinoma (hepatocellular), Magnetic resonance imaging, Prognosis, Hepatectomy

## Abstract

**Objectives:**

Contrast-enhanced MRI can provide individualized prognostic information for hepatocellular carcinoma (HCC). We aimed to investigate the value of MRI features to predict early (≤ 2 years)/late (> 2 years) recurrence-free survival (E-RFS and L-RFS, respectively) and overall survival (OS).

**Materials and methods:**

Consecutive adult patients at a tertiary academic center who received curative-intent liver resection for very early to intermediate stage HCC and underwent preoperative contrast-enhanced MRI were retrospectively enrolled from March 2011 to April 2021. Three masked radiologists independently assessed 54 MRI features. Uni- and multivariable Cox regression analyses were conducted to investigate the associations of imaging features with E-RFS, L-RFS, and OS.

**Results:**

This study included 600 patients (median age, 53 years; 526 men). During a median follow-up of 55.3 months, 51% of patients experienced recurrence (early recurrence: 66%; late recurrence: 34%), and 17% died. Tumor size, multiple tumors, rim arterial phase hyperenhancement, iron sparing in solid mass, tumor growth pattern, and gastroesophageal varices were associated with E-RFS and OS (largest *p* = .02). Nonperipheral washout (*p* = .006), markedly low apparent diffusion coefficient value (*p* = .02), intratumoral arteries (*p* = .01), and width of the main portal vein (*p* = .03) were associated with E-RFS but not with L-RFS or OS, while the VICT2 trait was specifically associated with OS (*p* = .02). Multiple tumors (*p* = .048) and radiologically-evident cirrhosis (*p* < .001) were the only predictors for L-RFS.

**Conclusion:**

Twelve visually-assessed MRI features predicted postoperative E-RFS (≤ 2 years), L-RFS (> 2 years), and OS for very early to intermediate-stage HCCs.

**Clinical relevance statement:**

The prognostic MRI features may help inform personalized surgical planning, neoadjuvant/adjuvant therapies, and postoperative surveillance, thus may be included in future prognostic models.

**Key Points:**

*• Tumor size, multiple tumors, rim arterial phase hyperenhancement, iron sparing, tumor growth pattern, and gastroesophageal varices predicted both recurrence-free survival within 2 years and overall survival.*

*• Nonperipheral washout, markedly low apparent diffusion coefficient value, intratumoral arteries, and width of the main portal vein specifically predicted recurrence-free survival within 2 years, while the VICT2 trait specifically predicted overall survival.*

*• Multiple tumors and radiologically-evident cirrhosis were the only predictors for recurrence-free survival beyond 2 years.*

**Graphical Abstract:**

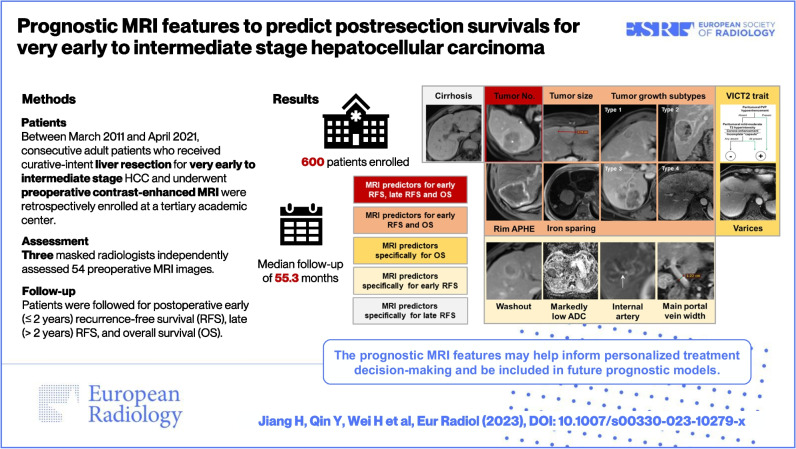

**Supplementary Information:**

The online version contains supplementary material available at 10.1007/s00330-023-10279-x.

## Introduction

Liver resection is the mainstay of curative-intent treatment for hepatocellular carcinoma (HCC) in patients without end-stage liver disease [1, 2], but postoperative recurrence or development of de novo HCC occurs in up to 50–70% of cases at 5 years [3–5].

Tumor stage based on tumor extension, liver function, and performance status are the key features for HCC prognostication. However, outcomes remain suboptimal despite patients being selected for resection based on these criteria. Likely, pathological and molecular characteristics (e.g., pathological subtype, tumor differentiation, microvascular invasion [MVI]) are additional prognostic factors [1, 3–6]. However, most of these features are only accessible on histopathological examinations, requiring assessment of the whole tumor/margin, and thus are only reliably available after surgery.

Fortunately, growing evidence suggested that key pathological and molecular characteristics of HCC may be inferred from imaging [7–14]. Among all imaging techniques, contrast-enhanced MRI is particularly suited for this task because it allows comprehensive evaluation of tumor morphology, hemodynamics, metabolism, and liver function via multiparametric imaging sequences [7, 8]. For example, non-smooth tumor margin, the two-trait predictor of venous invasion, hepatobiliary phase (HBP) peritumoral hypointensity, and its non-hepatobiliary-specific analogue (i.e., the VICT2 trait) have been associated with an increased risk of MVI [9–12]. Several of these and other MRI features have also been associated with survival outcomes after liver resection [15–18], highlighting the potential of MRI in profiling HCC aggressiveness. Despite promising results, prior data for MRI has been derived from studies with relatively small sample sizes, small numbers of prognostic features, analyses without control groups, and non-standardized treatments (e.g., unlimited tumor stage, unspecified use of adjuvant therapy) [15–18].

Therefore, this study aimed to systemically investigate the prognostic values, reliability, and clinical-radiological-pathological correlations for a total of 54 MRI features in patients who received curative-intent liver resection for very early to intermediate-stage HCCs.

## Materials and methods

This single-center retrospective cohort study was approved by the institutional review board at West China Hospital (approval number, 2022–1993) with a waiver of the informed consent.

### Patients

From March 2011 to April 2021, consecutive patients who fulfilled the following inclusion criteria were identified at an academic tertiary referral hospital: (**a**) age ≥ 18 years; (**b**) received curative-intent liver resection; (**c**) had pathologically-confirmed HCC; (**d**) with very early to intermediate stage tumors according to the 2022 Barcelona Clinic Liver Cancer (BCLC) staging system on preoperative MRI; and (**e**) underwent preoperative contrast-enhanced MRI within two months prior to surgery.

Patients were excluded if they: (**a**) received any previous treatment for HCC; (**b**) had any prior or current malignancy other than HCC; (**c**) had inadequate MR image quality for analyses (e.g., severe artifacts, incomplete sequences); (**d**) had ruptured HCC; (**e**) underwent contemporary ablation during surgery; (**f**) had BCLC C stage tumors on postoperative pathology; (**g**) underwent non-curative surgery [2]; (**h**) died from acute postoperative complications within 2 weeks; (**i**) received any adjuvant therapy; or (**j**) without follow-up information.

The resection extent and margin width were determined according to the surgeons’ discretion while considering patient performance status, liver function, estimated residual liver volume, tumor burden, and comorbidities [3]. Major resection was defined as resection of ≥ 3 segments according to the Couinaud classification, while minor resection was resection of < 3 segments [19]. All patients who met the Chinese Society of Hepatology criteria received antiviral therapy for hepatitis B as clinically indicated [20]. Post-recurrence treatments were discussed at the multidisciplinary tumor board.

Baseline clinical data (e.g., age, sex, etiologies of chronic liver diseases) and serum α-fetoprotein (AFP) within 14 days before surgery were recorded. Postoperative histopathologic data on well-established prognostic markers, including tumor differentiation (i.e., the lowest differentiation for being more prognostic), MVI, the macrotrabecular-massive (MTM) subtype, and cytokeratin 19 (CK19) expression for the largest tumor were retrieved from routine pathology reports.

### MRI acquisition protocols

The MR examinations were performed on various 1.5-T or 3.0-T MR systems. Either extracellular or hepatobiliary contrast agents were used. The MR sequences included standard liver protocol. Detailed MR acquisition protocols are presented in Supplemental Material 1.

### Image analysis

All deidentified MR images were reviewed independently by three fellowship-trained abdominal radiologists (R1, R2, and R3 with 7, 3, and 10 years of experience in liver MRI, respectively). A total of 200 randomly selected patients were assessed by R1 again after a one-month interval to evaluate the intra-observer agreement. The reviewers were aware that all patients had HCC but were blinded to the remaining clinical, pathological, and follow-up information.

On a per-patient basis, the reviewers evaluated 54 imaging features that have been reported to describe HCC or chronic liver diseases, including (**a**) tumor burden (e.g., tumor size and number), (**b**) the Liver Imaging Reporting and Data System (LI-RADS) v2018 features and categories [21], (**c**) other previously-reported tumor-related prognostic features (e.g., HBP peritumoral hypointensity, intratumoral arteries, nonsmooth tumor margin and the VICT2 trait) [8–12], and (**d**) features associated with the severity of underlying liver diseases and portal hypertension (e.g., radiologically-evident cirrhosis, gastroesophageal varices). All reviewers also assigned interpretation confidence of either high or low certainty for each feature. The largest tumor was selected for analyses in patients with multiple tumors. Detailed definitions of the imaging features are summarized in Supplemental Table 2. The difference between satellite tumors and the confluent multinodular type is graphically illustrated in Supplemental Fig. 1.

Disagreements on binary imaging features were resolved with the majority interpretations and those on ordinal/categorical imaging features by consulting a senior abdominal radiologist with over 20 years of experience in liver MRI.

### Patient follow-up

All patients underwent regular postoperative follow-ups at one month, every three months for the first two years, and every six months thereafter with serum AFP, ultrasound, contrast-enhanced CT, or MRI [3]. Bone scans and biopsies were performed if clinically indicated. Patients were followed until death or May 1, 2022.

Recurrence was defined as unequivocal radiological and/or histologic identification of intrahepatic HCC, tumor-in-vein, or distant metastasis, based on the diagnostic criteria of the American Association for the Study of Liver Diseases [4]. Recurrence was classified as early (occurring ≤ 2 years) or late (occurring > 2 years) after liver resection [4, 22, 23]. Recurrence-free survival was defined as the time from liver resection to first-documented tumor recurrence, or death of any cause, whichever occurred first. Therefore, early recurrence-free survival (E-RFS) and late recurrence-free survival (L-RFS) were separately analyzed. Specifically, E-RFS was assessed for all enrolled patients. Therefore, for patients who experienced recurrence or died ≤ 2 years after surgery, the survival endpoint would be positive for E-RFS, and the survival time would be the time from liver resection to recurrence or death. Contrarily, for those who were event-free ≤ 2 years after surgery, the survival endpoint would be negative for E-RFS, and the survival time would be the time from liver resection to the last available follow-up time (if ≤ 2 years) or 2 years (if > 2 years). By contrast, L-RFS was only assessed for patients who had at least 2 years of follow-up without recurrence or death within 2 years. Overall survival (OS) was defined as the time from liver resection to all-cause death.

### Statistical analysis

The sample size was estimated to ensure that at least ten outcome events per variable were available for effective multivariable Cox regression analyses [24].

The prognostic values of the imaging features were investigated as below. First, all imaging features were assessed for collinearity by Spearman’s correlation analysis and the variance inflation factors. Second, while controlling for patient age and sex, the prognostic values of all imaging features were assessed by univariable Cox regression analyses, and independent variables with *p* < 0.05 at the univariable analysis were input into a multivariable Cox regression model using the backward stepwise method. Survival outcomes were estimated by the Kaplan–Meier method and compared by the log-rank test. Subgroup analyses were conducted for clinical-pathological factors that impact patient survival, including BCLC stages (0 vs. A vs. B), serum AFP level (> 400 ng/mL vs. ≤ 400 ng/mL), resection extent (major vs. minor), resection margin width (≥ 10 mm vs. < 10 mm), tumor differentiation (poorly-differentiated vs. well-moderately differentiated), and MVI (present vs. absent).

Intra- and inter-observer agreements on continuous or ordinal/categorical imaging features were assessed with the intraclass correlation coefficient or the weighted kappa value, respectively. For binary variables, intra-observer agreements were evaluated with Cohen’s kappa value, while inter-observer agreements with Fleiss’ kappa value.

The statistical analyses were conducted using SPSS (version 25; IBM) or Medcalc (version 20.112; MedCalc Software). The Bonferroni method was used to adjust for multiple comparisons, and a two-tailed *p* < 0.05 was considered statistically significant.

## Results

### Patients

The patient characteristics are summarized in Table 1.

**Table 1:** Clinical-pathologic characteristics of the enrolled patients

Characteristics	
Patient factors	
Age, yr	53 (45–61)
Gender	
Male	526 (88)
Female	74 (12)
Underlying liver diseases	
Hepatitis B virus	568 (95)
Hepatitis C virus	7 (1)
Hepatitis B and C virus coinfection	8 (1)
Others	17 (3)
Child–Pugh score	
A	566 (94)
B	34 (6)
Serum AFP*	
> 400 ng/mL	150 (25)
≤ 400 ng/mL	446 (75)
Pathology-confirmed cirrhosis	335 (56)
Tumor factors	
Tumor size, cm	3.3 (2.3–5.1)
Tumor number	
Solitary	530 (88)
2–3 tumors	61 (10)
Over 3 tumors	9 (2)
Barcelona Clinic Liver Cancer Stage	
0	103 (17)
A	445 (74)
B	52 (9)
Tumor differentiation*	
Poorly differentiated tumors	181 (31)
Well-moderately differentiated tumors	404 (69)
Microvascular invasion*	
Present	104 (35)
Absent	196 (65)
Cytokeratin 19 expression *	
Positive	27 (15)
Negative	148 (85)
Surgical factors	
Intraoperative blood loss, mL	200 (50–300)
Intraoperative blood transfusion	23 (4)
Resection extent*	
Major	98 (17)
Minor	479 (83)
Resection margin width*	
≥ 10 mm	93 (29)
< 10 mm	231 (71)
Operation time*	
> 3 h	187 (44)
≤ 3 h	241 (56)

Unless stated otherwise, data in parentheses are interquartile ranges or percentages

^*^Data are presented for patients who had complete documentation on these factors

*AFP* α-fetoprotein; *CK19* cytokeratin 19

**Fig. 1:**
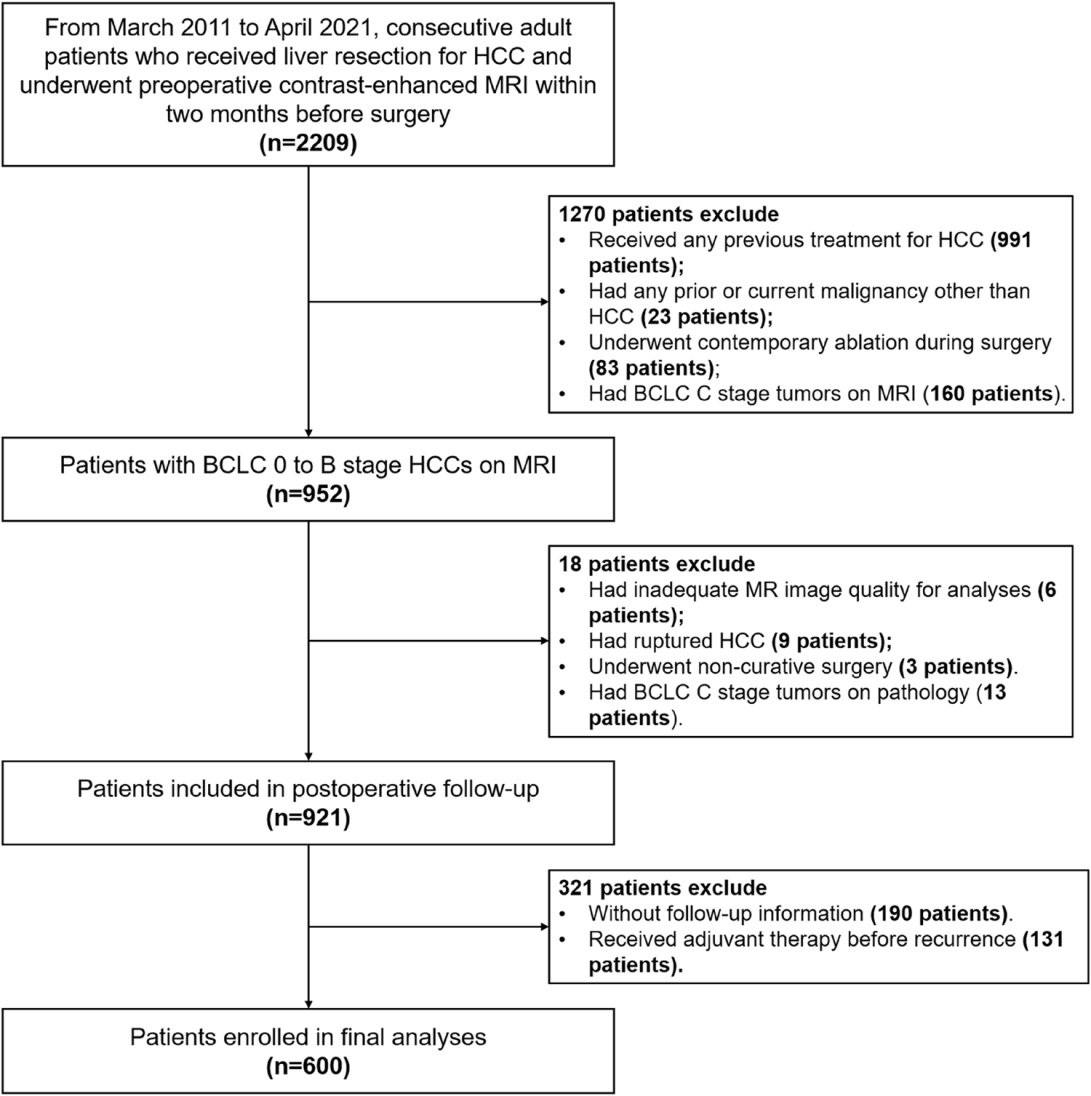
Study flowchart. *HCC*, hepatocellular carcinoma

A total of 600 patients (median age, 53 years; interquartile range [IQR], 45–61 years; 526 [87.7%] men) were included (Fig. 1), 95% (568/600) of them had chronic hepatitis B, and 56% (335/600) had pathologically-confirmed cirrhosis. Up to 70% (417/600) of patients underwent extracellular contrast agent-enhanced MRI, while 30% (183/600) underwent hepatobiliary contrast agent-enhanced MRI.

Solitary tumors were observed in 88% (530/600) of patients, and the median size was 3.3 cm (IQR, 2.3–5.1 cm). A total of 17% (103/600), 74% (445/600), and 9% (52/600) of patients had BCLC 0, A, and B stage disease, respectively. Poorly-differentiated tumors, MVI, the MTM subtype, and CK19-positive tumors were present in 31% (181/585), 35% (104/300), 18% (36/195), and 15% (27/175) of patients, respectively. Major resection was performed in 17% (98/577) of patients, and 71% (231/324) had resection margins of < 10 mm. A total of 44% (187/428) of patients had operations lasting over 3 h, and 4% (23/600) required blood transfusion during the perioperative period.

The median follow-up was 55.3 months (IQR, 41.0–76.8 months). During this period, 51% (307/600) of patients experienced recurrence (median RFS, 51.2 months; 95%CI, 41.8–59.4 months). Among them, 66% (204/307) of patients developed early recurrence, while 34% (103/307) experienced late recurrence. Additionally, 17% (102/600) of patients died (median survival, not reached); the 1-year, 3-year, and 5-year OS rates were 98%, 89%, and 79%, respectively.

### Identification of prognostic imaging features

Because only one patient (0.2%) had HCC with the infiltrative type, the infiltrative type was grouped together with the confluent multinodular type for all further analyses.

#### Imaging predictors for E-RFS (≤ 2 years)

Thirty-one imaging features were associated with E-RFS at univariable Cox regression analyses, and 18 were retained after accounting for collinearity (Table 2). Among them, multiple tumors (HR, 2.1; 95%CI: 1.4–2.9; *p* < 0.001), tumor size (for every 1 cm increase; HR, 1.1; 95%CI: 1.0–1.2; *p* < 0.001), nonperipheral washout (HR, 1.7; 95%CI: 1.1–2.5; *p* = 0.006), rim APHE (HR, 2.6; 95%CI, 1.6–4.3; *p* < 0.001), iron sparing in solid mass (HR, 1.7; 95% CI: 1.2–2.4; *p* = 0.002), markedly low apparent diffusion coefficient value (HR, 1.7; 95% CI: 1.1–2.6; *p* = 0.02), intratumoral artery (HR, 1.6; 95% CI: 1.1–2.4; *p* = 0.01), confluent multinodular or infiltrative type (HR, 2.7; 95%CI, 1.3–5.8; *p* = 0.01), the width of the main portal vein (for every 1 cm increase; HR, 1.7; 95%CI: 1.0–2.8; *p* = 0.03), and gastroesophageal varices (HR, 1.4; 95%CI, 1.0–1.8; *p* = 0.02) were associated with E-RFS at the multivariable Cox regression analysis.

**Table 2:** Imaging predictors for early (≤ 2 years) and late (> 2 years) recurrence-free survival

Imaging features	Early recurrence-free survival				Late recurrence-free survival			
	Univariable HR	*p value*	Multivariable HR*	*p value*	Univariable HR	*p value*	Multivariable HR†	*p value*
LI-RADS major features								
Nonrim arterial phase hyperenhancement (present vs. absent)	0.6 (0.4–0.9)	***.02***	…	*…*	…	*…*	…	*…*
Nonperipheral washout (present vs. absent)	1.6 (1.1–2.3)	***.01***	1.7 (1.1–2.5)	***.006***	…	*…*	…	*…*
Tumor size (cm)	1.2 (1.1–1.2)	** < *.001***	1.1 (1.0–1.2)	** < *.001***	…	…	…	…
LI-RADS ancillary features								
Corona enhancement (present vs. absent)	1.4 (1.0–1.9)	***.02***	…	*…*	…	*…*	…	*…*
Mosaic architecture (present vs. absent)	2.4 (1.8–3.3)	** < *.001***	…	*…*	…	*…*	…	*…*
Blood products in mass (present vs. absent)	2.4 (1.8–3.1)	** < *.001***	…	*…*	…	*…*	…	*…*
Iron sparing in solid mass (present vs. absent)	1.9 (1.4–2.7)	** < *.001***	1.7 (1.2–2.4)	***.002***	…	*…*	…	*…*
LR-M features								
Rim arterial phase hyperenhancement (present vs. absent)	2.6 (1.7–4.0)	** < *.001***	2.6 (1.6–4.3)	** < *.001***	…	*…*	…	*…*
Marked diffusion restriction (present vs. absent)	1.5 (1.1–2.1)	***.008***	…	*…*	…	*…*	…	*…*
Infiltrative appearance (present vs. absent)	3.3 (2.0–5.4)	** < *.001***	…	*…*	…	*…*	…	*…*
Necrosis or severe ischemia (present vs. absent)	2.1 (1.6–2.8)	** < *.001***	…	*…*	…	*…*	…	*…*
LI-RADS category								
LR-4	*Ref*	*…*	…	*…*	*…*	*…*	…	*…*
LR-5	…	*…*	…	*…*	…	*…*	…	*…*
LR-M	2.2 (1.4–3.5)	** < *.001***	…	*…*	…	*…*	…	*…*
LI-RADS M category (present vs. absent)	2.2 (1.4–3.5)	** < *.001***	…	*…*	…	*…*	…	*…*
Other tumor-related prognostic features								
Mild-to-moderate T2-weighted peritumoral hyperintensity (present vs. absent)	2.1 (1.5–2.8)	** < *.001***	…	*…*	…	*…*	…	*…*
Portal venous phase peritumoral hypoenhancement (present vs. absent)	2.5 (1.8–3.3)	** < *.001***	…	*…*	…	*…*	…	*…*
Markedly low apparent diffusion coefficient value (present vs. absent)	2.2 (1.5–3.3)	** < *.001***	1.7 (1.1–2.6)	***.02***	…	*…*	…	*…*
≥ 50% arterial phase hyperenhancement (present vs. absent)	0.6 (0.5–0.8)	***.001***	…	*…*	…	*…*	…	*…*
Intratumoral arteries (present vs. absent)	2.7 (2.0–3.6)	** < *.001***	1.6 (1.1–2.4)	***.01***	…	*…*	…	*…*
Complete capsule (present vs. absent)	0.5 (0.3–0.7)	** < *.001***	…	*…*	…	*…*	…	*…*
Non-smooth tumor margin (present vs. absent)	2.0 (1.4–2.8)	** < *.001***	…	*…*	…	*…*	…	*…*
The VICT2 trait (present vs. absent)	2.3 (1.7–3.1)	** < *.001***	…	*…*	…	*…*	…	*…*
The two-trait predictor of venous invasion (present vs. absent)	2.6 (1.9–3.4)	** < *.001***	…	*…*	…	*…*	…	*…*
Tumor growth subtype								
Single nodular type	*Ref*	*…*	*Ref*	*…*	*…*	*…*	*…*	*…*
Single nodule type with extranodular growth	1.8 (1.3–2.3)	** < *.001***	…	*…*			…	*…*
Confluent multinodular or infiltrative type	7.5 (3.6–15.5)	** < *.001***	2.7 (1.3–5.8)	***.01***	…	*…*	…	*…*
Imaging features associated with tumor burden								
Tumor number								
Solitary	*Ref*	*…*	…	*…*	*Ref*	*…*	…	*…*
2–3 tumors	2.2 (1.5–3.3)	** < *.001***	…	*…*	2.0 (1.1–3.6)	***.03***	…	*…*
Over 3 tumors	3.6 (1.7–7.3)	** < *.001***	…	*…*	…	*…*	…	*…*
Tumor number (solitary vs. multiple)	2.4 (1.7–3.4)	** < *.001***	2.1 (1.4–2.9)	** < *.001***	2.2 (1.2–4.0)	***.01***	2.0 (1.3–5.8)	***.048***
Satellite tumors (present vs. absent)	2.0 (1.1–3.4)	***.02***	…	*…*	…	*…*	…	*…*
Imaging features associated with the severity of underlying liver diseases and portal hypertension								
Radiologically-evident cirrhosis (present vs. absent)	…	*…*	…	*…*	2.8 (1.7–4.7)	** < *.001***	2.7 (1.7–4.6)	** < *.001***
Diffuse iron overload (present vs. absent)	1.6 (1.2–2.3)	***.002***	…	*…*	…	*…*	…	*…*
Diffuse fatty change (present vs. absent)	…	*…*	…	*…*	…	*…*	…	*…*
Width of the main portal vein (cm)	1.9 (1.3–2.9)	***.003***	1.7 (1.0–2.8)	***.03***	…	*…*	…	*…*
Splenomegaly (present vs. absent)	1.4 (1.1–1.9)	***.02***	…	*…*	1.6 (1.1–2.3)	***.03***	…	*…*
Porto-systemic shunts (present vs. absent)	…	*…*	…	*…*	…	*…*	…	*…*
Gastroesophageal varices (present vs. absent)	1.5 (1.1–1.9)	***.006***	1.4 (1.0–1.8)	***.02***	…	*…*	…	*…*

Data are presented only for imaging features which are associated with survival outcomes at the univariable Cox regression analyses. Unless stated otherwise, data in parentheses are 95% confidence intervals. All *p* values < .05 are highlighted in bold

^*^To minimize over-fitting, variables independent of collinearity with *p* values < .05 at univariable Cox regression analysis (*n* = 18) were input into the multivariable Cox regression model with stepwise method while controlling for patient age and sex, including nonperipheral washout, tumor size, corona enhancement, mosaic architecture, blood products in mass, iron sparing in solid mass, rim arterial phase hyperenhancement, infiltrative appearance, mild to moderate T2-weighted peritumoral hyperintensity, portal venous phase peritumoral hypoenhancement, markedly low apparent diffusion coefficient value, ≥ 50% arterial phase hyperenhancement, intratumoral arteries, complete capsule, tumor growth subtype, tumor number (solitary vs. multiple), width of main portal vein (cm), and gastroesophageal varices

Hepatobiliary-specific imaging features (i.e., imaging features measurable on transitional or hepatobiliary phase images) were not evaluated in the multivariable analysis because these measurements were only available for patients who underwent hepatobiliary contrast agent -enhanced MRI (*n* = 183)

^†^Variables independent of collinearity with *p* values < .05 at univariable Cox regression analysis (*n* = 3) were input into the multivariable Cox regression model with stepwise method while controlling for patient age and sex, including tumor number (solitary vs. multiple), radiologically-evident cirrhosis, and splenomegaly

*HR* = hazard ratio; *LI-RADS* = Liver Imaging Reporting and Data System

#### Imaging predictors for L-RFS (> 2 years)

A total of four imaging features were associated with L-RFS at univariable Cox regression analyses, and three were retained after accounting for collinearity (Table 2). Among them, multiple tumors (HR, 2.0; 95%CI: 1.3–5.8; *p* = 0.048) and radiologically-evident cirrhosis (HR, 2.7; 95%: 1.7–4.6; *p* < 0.001) were associated with L-RFS at the multivariable Cox regression analysis.

**Fig. 2:**
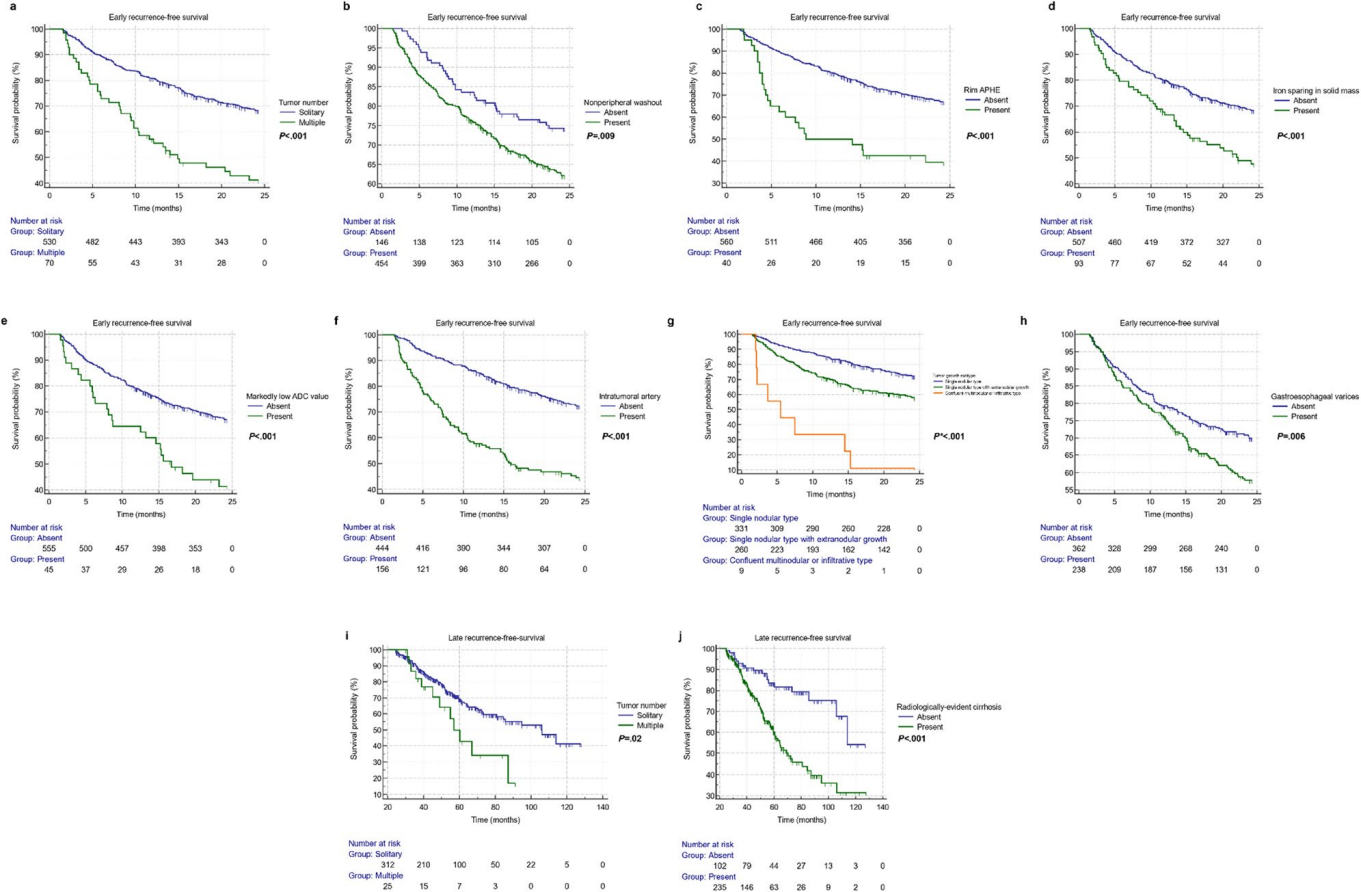
Kaplan–Meier curves of the binary/ordinal prognostic imaging features for early (≤ 2 years, **a**–**h**) and late (> 2 years, **i** and **j**) recurrence-free survival. *The *p* value was computed after correction for multiple comparisons with the Bonferroni method. APHE, arterial phase hyperenhancement

E-RFS and L-RFS outcomes are plotted in Fig. 2.

#### Imaging predictors for OS

A total of 29 imaging features were associated with OS at univariable Cox regression analyses, and ten were retained after accounting for collinearity (Table 3). Among them, multiple tumors (HR, 1.9; 95%CI: 1.2–3.1; *p* = 0.007), tumor size (for every 1 cm increase; HR, 1.1; 95%CI: 1.1–1.2; *p* < 0.001), rim APHE (HR, 3.3; 95% CI: 1.9–5.6; *p* < 0.001), iron sparing in solid mass (HR, 2.3; 95% CI: 1.4–3.6; *p* < 0.001), the VICT2 trait (HR, 1.8; 95%CI, 1.1–2.8; *p* = 0.02), tumor growth subtype (single nodule type with extranodular growth: HR, 1.7; 95% CI, 1.1–2.6; *p* = 0.02; confluent multinodular or infiltrative type: HR, 4.2; 95%CI, 1. 6–10.6; *p* = 0.003) and gastroesophageal varices (HR, 1.8; 95% CI, 1.2–2.7; *p* = 0.004) were associated with OS at the multivariable Cox regression analysis.

**Table 3:** Imaging predictors for overall survival

Imaging features	Univariable HR	*p value*	Multivariable HR*	*p value*
LI-RADS major features				
Nonrim arterial phase hyperenhancement (present vs. absent)	0.4 (0.2–0.6)	** < *.001***	…	*…*
Tumor size (cm)	1.2 (1.1–1.3)	** < *.001***	1.1 (1.1–1.2)	** < *.001***
LI-RADS ancillary features				
Corona enhancement (present vs. absent)	2.0 (1.4–3.0)	** < *.001***	…	*…*
Nodule-in-nodule architecture (present vs. absent)	1.6 (1.0–2.5)	***.04***	…	*…*
Mosaic architecture (present vs. absent)	2.7 (1.8–4.0)	** < *.001***	…	*…*
Blood products in mass (present vs. absent)	2.6 (1.8–3.9)	** < *.001***	…	*…*
Iron sparing in solid mass (present vs. absent)	2.6 (1.7–4.0)	** < *.001***	2.3 (1.4–3.6)	** < *.001***
LR-M features				
Rim arterial phase hyperenhancement (present vs. absent)	4.1 (2.4–6.8)	** < *.001***	3.3 (1.9–5.6)	** < *.001***
Infiltrative appearance (present vs. absent)	4.3 (2.3–8.0)	** < *.001***	…	*…*
Necrosis or severe ischemia (present vs. absent)	2.4 (1.7–3.6)	** < *.001***	…	*…*
LI-RADS category				
LR-4	*Ref*	*…*	…	*…*
LR-5	…	*…*	…	*…*
LR-M	3.4 (2.0–5.8)	** < *.001***	…	*…*
LI-RADS M category (present vs. absent)	3.5 (2.1–6.0)	** < *.001***	…	*…*
Other tumor-related prognostic features				
Mild-to-moderate T2-weighted peritumoral hyperintensity (present vs. absent)	2.8 (1.8–4.2)	** < *.001***	…	*…*
Portal venous phase peritumoral hypoenhancement (present vs. absent)	2.9 (1.9–4.4)	** < *.001***	…	*…*
Markedly low apparent diffusion coefficient value (present vs. absent)	2.0 (1.1–3.5)	***.02***	…	*…*
≥ 50% arterial phase hyperenhancement (present vs. absent)	0.5 (0.3–0.7)	** < *.001***	…	*…*
Intratumoral arteries (present vs. absent)	2.7 (1.8–4.0)	** < *.001***	…	*…*
Complete capsule (present vs. absent)	0.4 (0.2–0.7)	***.001***	…	*…*
Non-smooth tumor margin (present vs. absent)	2.9 (1.6–5.2)	** < *.001***	…	*…*
HBP peritumoral hypointensity (present vs. absent)†	3.1 (1.3–7.6)	***.01***	…	*…*
The VICT2 trait (present vs. absent)	3.0 (2.0–4.5)	** < *.001***	1.8 (1.1–2.8)	***.02***
The two-trait predictor of venous invasion (present vs. absent)	2.5 (1.7–3.7)	** < *.001***	…	*…*
Tumor growth subtype				
Single nodular type	*Ref*	*…*	*Ref*	*…*
Single nodule type with extranodular growth	2.5 (1.6–3.7)	** < *.001***	1.7 (1.1–2.6)	***.02***
Confluent multinodular or infiltrative type	12.3 (5.1–29.4)	** < *.001***	4.2 (1.6–10.6)	***.003***
Imaging features associated with tumor burden				
Tumor number				
Solitary	*Ref*	*…*	…	*…*
2–3 tumors	2.6 (1.5–4.3)	** < *.001***	…	*…*
Over 3 tumors	6.4 (2.9–13.9)	** < *.001***	…	*…*
Tumor number (solitary vs. multiple)	3.1 (2.0–4.9)	** < *.001***	1.9 (1.2–3.1)	***.007***
Satellite tumors (present vs. absent)	2.8 (1.4–5.3)	***.002***	…	*…*
Imaging features associated with the severity of underlying liver diseases and portal hypertension				
Diffuse iron overload (present vs. absent)	2.1 (1.4–3.4)	** < *.001***	…	*…*
Width of main portal vein (cm)	2.2 (1.2–3.8)	***.008***	…	*…*
Gastroesophageal varices (present vs. absent)	1.7 (1.2–2.5)	***.006***	1.8 (1.2–2.7)	***.004***

Data are presented only for imaging features which are associated with survival outcomes at the univariable Cox regression analyses. Unless stated otherwise, data in parentheses are 95% confidence intervals. All* p *values < .05 are highlighted in bold

^*^To minimize over-fitting, variables independent of collinearity with *p* values < .05 at univariable Cox regression analysis (*n* = 10) were input into the multivariable Cox regression model with a stepwise method while controlling for patient age and sex. These variables included tumor size, iron sparing in solid mass, rim arterial phase hyperenhancement, infiltrative appearance, ≥ 50% arterial phase hyperenhancement, intratumoral arteries, the VICT2 trait, tumor growth subtype, tumor number (solitary vs. multiple), and gastroesophageal varices. Hepatobiliary-specific imaging features (i.e., imaging features measurable on transitional or hepatobiliary phase images) were not evaluated in the multivariable analysis because these measurements were only available for patients who underwent hepatobiliary contrast agent -enhanced MRI (*n* = 183)

^†^Analyses were conducted in patients who underwent hepatobiliary contrast agent-enhanced MRI (*n* = 183)

*HR* = hazard ratio; *LI-RADS* = Liver Imaging Reporting and Data System; *HBP* = hepatobiliary phase

**Fig. 3:**
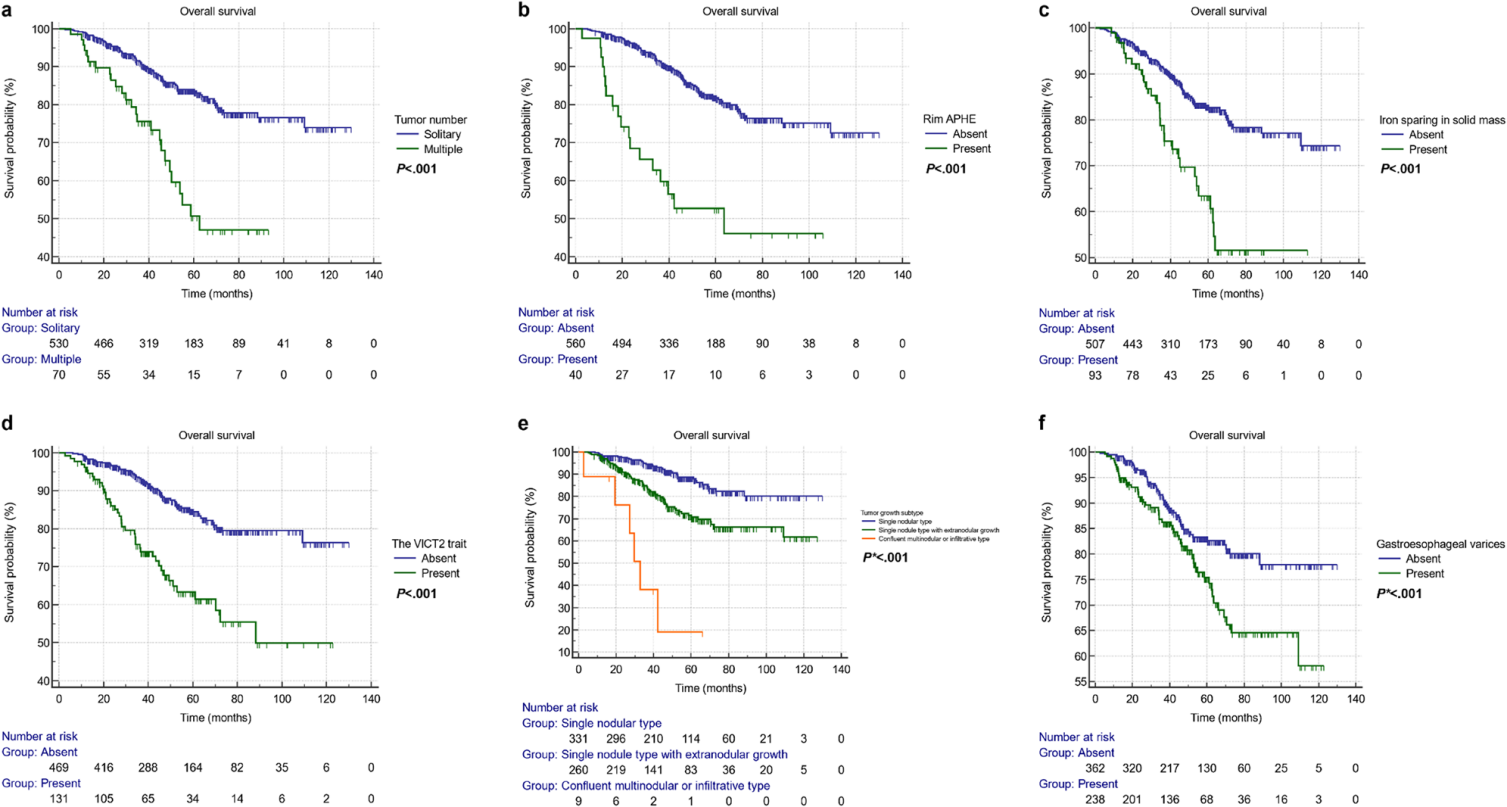
Kaplan–Meier curves of the binary/ordinal prognostic imaging features for overall survival. The VICT2 trait is considered present when peritumoral PVP hypoenhancement is present or if corona enhancement, peritumoral mild-moderate hypointensity, and incomplete capsule are all present; otherwise, negative. *The *p* value was computed after correction for multiple comparisons with the Bonferroni method. APHE, arterial phase hyperenhancement

OS outcomes are plotted in Fig. 3.

#### Subgroup analyses

E-RFS and OS were distinct for all subgroups (*p* values, < 0.001 to 0.04); however, no difference in L-RFS was observed for any of these subgroups (*p* values, 0.36 to 0.86). Therefore, subgroup analyses were not performed for L-RFS.

Multiple tumors were associated with worse E-RFS and OS in most subgroups, except for patients undergoing major resection (RFS, *p* = 0.38) and in subgroups stratified with MVI (*p* values, 0.09 to 0.62). Tumor size was associated with E-RFS in most subgroups, except for patients with BCLC 0 tumors (*p* = 0.62). Rim APHE was associated with worse OS in all subgroups and with worse E-RFS in most subgroups except for patients who underwent major resection (*p* = 0.15) and those with MVI (*p* = 0.09). Iron sparing in solid mass was associated with worse E-RFS in most subgroups, except for patients with BCLC 0 stage tumors (*p* = 0.19) and in subgroups stratified with MVI (*p* values, 0.05 to 0.56); similarly, it was also associated with worse OS in most subgroups, except for patients with BCLC B stage tumors (*p* = 0.28) and in patients with MVI (*p* = 0.10).

Noteworthily, in patients with BCLC 0 stage tumors (*n* = 103), gastroesophageal varices (*p* = 0.004) and the width of the main portal vein (*p* = 0.02) were the only imaging markers linked to E-RFS, while the presence of gastroesophageal varices was the only imaging marker associated with OS (*p* = 0.045).

Subgroup analyses of other prognostic imaging features are detailed as forest plots in Supplemental Figs. 2–18.

### Frequencies and agreement of prognostic imaging features

The frequencies of the above prognostic imaging features ranged from 1% (9/600) for confluent multinodular or infiltrative subtypes to 76% (454/600) for nonperipheral washout.

For qualitative imaging features, intra-observer agreement ranged from fair for iron sparing in solid mass (Cohen’s κ value, 0.27; 95%CI, 0.07–0.47) to substantial for nonperipheral washout (Cohen’s κ value, 0.79; 95%CI, 0.70–0.88), while inter-observer agreement ranged from poor for iron sparing in solid mass (Fleiss κ value, − 0.01; 95%CI, − 0.06 to 0.04) to substantial for tumor number (Fleiss κ value, 0.69; 95%CI, 0.64–0.74). For tumor size, both intra- (intraclass correlation coefficient [ICC], 0.99; 95%CI, 0.99–0.99) and inter-observer (ICC, 0.98; 95%CI, 0.97–0.98) agreement were excellent. For the width of the main portal vein, the intra-observer agreement was excellent (ICC, 0.92; 95%CI, 0.89–0.94), while the inter-observer agreement was substantial (ICC, 0.69; 95%CI, 0.65–0.72).

**Fig. 4:**
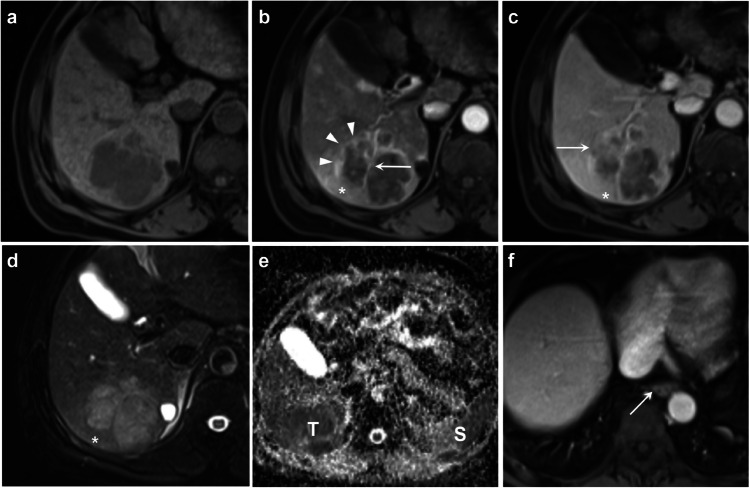
Contrast-enhanced MR images of a 61-year-old male patient who had chronic hepatitis B. A 7.1 cm mass with confluent multinodular growth type was detected in segment VI and VII. The mass shows hypointensity on T1-weighted pre-contrast images (**a**), rim arterial phase hyperenhancement (*arrowheads*), and the presence of intratumoral arteries (*arrow*) on arterial phase images (**b**), mild-moderate T2 hyperintensity on T2-weighted images (**d**), mild-moderately low apparent diffusion correlation value of the tumor (***T***) in relative to the spleen (***S***) (**e**), and the presence of gastroesophageal varices (*arrow*,** f**). The mass also demonstrates corona enhancement (*star*, **b**), subtle portal venous phase peritumoral hypoenhancement (*star*, **c**), incomplete capsule (*arrow*,** c**), and mild-moderate T2-weighted peritumoral hyperintensity (*star*, **d**), corresponding to the presence of the VICT2 trait. The mass was histopathologically confirmed as a poorly-differentiated HCC with microvascular invasion. The recurrence-free survival and overall survival of this patient was 60 days and 80 days, respectively

Contrast-enhanced MR images of a typical case are shown in Fig. 4. The frequencies, interpretation certainties, and agreements on all imaging features are presented in Table 4.

**Table 4:** Frequencies, interpretation certainties, and agreement of the all evaluated MRI features

Imaging features	Frequency				High certainty interpretation			Agreement*	
	Consensus	R1	R2	R3	R1	R2	R3	Intra-observer	Inter-observer
LI-RADS major features									
Nonrim arterial phase hyperenhancement (present vs. absent)	552 (92)	551 (92)	554 (92)	527 (88)	540 (90)	560 (93)	567 (95)	0.535 (0.250–0.820)	0.501 (0.455–0.547)
Nonperipheral washout (present vs. absent)	454 (76)	454 (76)	454 (76)	446 (74)	542 (90)	566 (94)	565 (94)	0.793 (0.702–0.884)	0.535 (0.489–0.581)
Enhancing capsule (present vs. absent)	529 (88)	523 (87)	524 (87)	477 (80)	510 (85)	568 (95)	568 (95)	0.528 (0.312–0.743)	0.431 (0.385–0.477)
Tumor size (cm)	…	…	…	…	595 (99)	600 (100)	599 (99.8)	0.989 (0.986–0.992)	0.978 (0.974–0.981)
LI-RADS ancillary features									
Diffusion restriction (present vs. absent)	600 (100)	598 (99.6)	592 (99)	598 (99.6)	588 (98)	589 (98)	588 (98)	− 0.005 (–0.012–0.002)	− 0.007 (− 0.053–0.039)
Mild-moderate T2 hyperintensity (present vs. absent)	589 (98)	585 (98)	593 (99)	567 (95)	585 (98)	594 (99)	590 (98)	0.236 (–0.164–0.636)	0.250 (0.204–0.296)
Corona enhancement (present vs. absent)	158 (26)	209 (35)	85 (14)	184 (31)	497 (83)	557 (93)	544 (91)	0.593 (0.472–0.713)	0.259 (0.213–0.306)
Nonenhancing capsule (present vs. absent)	21 (4)	26 (4)	35 (6)	32 (5)	592 (99)	596 (99)	590 (98)	0.664 (0.046–1.000)	0.478 (0.432–0.525)
Nodule-in-nodule architecture (present vs. absent)	127 (21)	209 (35)	77 (13)	124 (21)	491 (82)	547 (91)	572 (95)	0.698 (0.589–0.807)	0.353 (0.306–0.399)
Mosaic architecture (present vs. absent)	141 (24)	146 (24)	115 (19)	182 (30)	527 (88)	554 (92)	575 (96)	0.616 (0.480–0.752)	0.515 (0.469–0.561)
Blood products in mass (present vs. absent)	139 (23)	161 (27)	121 (20)	144 (24)	549 (92)	578 (96)	576 (96)	0.755 (0.637–0.873)	0.689 (0.643–0.736)
Fat in mass, more than adjacent liver (present vs. absent)	179 (30)	234 (39)	205 (34)	140 (23)	500 (83)	531 (89)	568 (95)	0.532 (0.411–0.653)	0.450 (0.404–0.496)
Fat sparing in solid mass (present vs. absent)	35 (6)	28 (5)	268 (45)	43 (7)	550 (92)	565 (94)	589 (98)	0.419 (0.125–0.714)	0.019 (− 0.027–0.065)
Iron sparing in solid mass (present vs. absent)	93 (16)	89 (15)	394 (66)	64 (11)	518 (86)	590 (98)	572 (95)	0.266 (0.065–0.467)	− 0.008 (–0.055–0.038)
TP hypointensity (preset vs. absent)†	175 (96)	174 (95)	176 (96)	169 (92)	180 (98)	181 (99)	182 (99)	0.486 (− 0.125–1.000)	0.506 (0.423–0.59)
HBP hypointensity (preset vs. absent)†	177 (97)	176 (96)	179 (98)	173 (95)	164 (90)	173 (95)	179 (98)	0.486 (− 0.125–1.000)	0.554 (0.471–0.638)
Marked T2 hyperintensity (present vs. absent)	10 (2)	14 (2)	6 (1)	33 (6)	589 (98)	596 (99)	596 (99)	0.393 (− 0.154–0.940)	0.281 (0.235–0.327)
Iron in mass, more than liver (present vs. absent)	2 (0.3)	2 (0.3)	7 (1)	18 (3)	558 (93)	597 (99.5)	585 (98)	…	0.060 (0.014–0.106)
Parallels blood pool enhancement (present vs. absent)	2 (0.3)	2 (0.3)	0 (0)	3 (0.5)	600 (100)	600 (100)	597 (99.5)	…	0.398 (0.352–0.445)
Undistorted vessels (present vs. absent)	0 (0)	0 (0)	0 (0)	2 (0.3)	600 (100)	600 (100)	598 (99.6)	…	− 0.001 (–0.047–0.045)
LR-M features									
Rim arterial phase hyperenhancement (present vs. absent)	40 (7)	40 (7)	41 (7)	55 (9)	567 (95)	579 (97)	584 (97)	0.535 (0.250–0.820)	0.515 (0.469–0.561)
Peripheral "washout" (present vs. absent)	7 (1)	8 (1)	12 (2)	22 (4)	593 (99)	600 (100)	594 (99)	…	0.196 (0.149–0.242)
Delayed central enhancement (present vs. absent)	7 (1)	9 (2)	9 (2)	22 (4)	595 (99)	596 (99)	584 (97)	0.745 (0.404–1.000)	0.259 (0.212–0.305)
Targetoid restriction (present vs. absent)	12 (2)	11 (2)	22 (4)	24 (4)	598 (99.6)	596 (99)	589 (98)	0.495 (–0.109–1.000)	0.402 (0.356–0.448)
Targetoid TP or HBP appearance (present vs. absent)†	3 (2)	2 (1)	4 (2)	4 (2)	182 (99)	181 (99)	182 (99)	1.000 (1.000–1.000)	0.491 (0.407–0.574)
Marked diffusion restriction (present vs. absent)	119 (20)	93 (16)	270 (45)	123 (21)	562 (94)	574 (96)	580 (97)	0.498 (0.315–0.681)	0.315 (0.269–0.361)
Infiltrative appearance (present vs. absent)	25 (4)	22 (4)	140 (23)	31 (5)	585 (98)	580 (97)	583 (97)	–0.020 (–0.034 to –0.006)	0.118 (0.072–0.164)
Necrosis or severe ischemia (present vs. absent)	184 (31)	202 (34)	139 (23)	209 (35)	542 (90)	574 (96)	585 (98)	0.701 (0.586–0.816)	0.649 (0.603–0.695)
LI-RADS category									
LR-3	0 (0)	0 (0)	0 (0)	12 (2)	596 (99)	597 (99.5)	578 (96)	0.527 (0.368–0.687)	0.850 (0.834–0.870)
LR-4	60 (10)	64 (11)	58 (10)	80 (13)					
LR-5	504 (84)	496 (83)	506 (84)	459 (77)					
LR-M	36 (6)	40 (7)	36 (6)	49 (8)					
LI-RADS M category (present vs. absent)	38 (6)	40 (7)	36 (6)	49 (8)	596 (99)	597 (99.5)	578 (96)	0.503 (0.222–0.785)	0.544 (0.498–0.591)
Other tumor-related prognostic features									
Pre-contrast T1-weighted hypointensity (present vs. absent)	572 (95)	575 (96)	500 (83)	559 (93)	592 (99)	598 (99.6)	598 (99.6)	0.516 (0.202–0.829)	0.204 (0.157–0.250)
T2-weighted peritumoral hyperintensity (present vs. absent)	95 (16)	109 (18)	128 (21)	86 (14)	559 (93)	570 (95)	584 (97)	0.524 (0.375–0.673)	0.479 (0.433–0.526)
Portal venous phase peritumoral hypoenhancement (present vs. absent)	101 (17)	118 (20)	115 (19)	102 (17)	550 (92)	576 (96)	575 (96)	0.615 (0.466–0.765)	0.545 (0.499–0.591)
Markedly low apparent diffusion coefficient value (present vs. absent)	45 (8)	38 (6)	234 (39)	44 (7)	566 (94)	568 (95)	566 (94)	0.451 (0.182–0.720)	0.067 (0.021–0.113)
≥ 50% arterial phase hyperenhancement (present vs. absent)	459 (77)	446 (74)	507 (85)	446 (74)	514 (86)	569 (95)	556 (93)	0.742 (0.636–0.848)	0.525 (0.479–0.571)
Intratumoral artery (present vs. absent)	156 (26)	144 (24)	203 (34)	164 (27)	513 (86)	561 (94)	563 (94)	0.743 (0.628–0.858)	0.516 (0.470–0.563)
Complete "capsule" (present vs. absent)	159 (27)	171 (28)	197 (33)	155 (26)	504 (84)	561 (94)	562 (94)	0.563 (0.442–0.684)	0.321 (0.275–0.367)
Non-smooth tumor margin (present vs. absent)	431 (72)	420 (71)	442 (74)	381 (64)	510 (85)	566 (94)	582 (97)	0.516 (0.390–0.642)	0.475 (0.429–0.521)
Marked HBP hypointensity (present vs. absent)†	112 (61)	90 (49)	117 (64)	124 (68)	164 (90)	173 (95)	179 (98)	0.530 (0.319–0.740)	0.399 (0.315–0.483)
HBP peritumoral hypointensity (present vs. absent)†	73 (40)	78 (43)	75 (41)	66 (36)	159 (87)	166 (91)	171 (93)	0.715 (0.552–0.879)	0.727 (0.643–0.810)
The VICT2 trait (present vs. absent)	131 (22)	159 (27)	132 (22)	131 (22)	…	…	…	0.544 (0.405–0.683)	0.502 (0.455–0.548)
The TTPVI trait (present vs. absent)	149 (25)	136 (23)	188 (31)	155 (26)	…	…	…	0.743 (0.628–0.858)	0.502 (0.456–0.548)
Tumor growth subtype									
Single nodular type	331 (55)	267 (45)	406 (68)	346 (58)	526 (88)	576 (96)	588 (98)	0.558 (0.446–0.670)	0.722 (0.696–0.754)
Single nodule type with extranodular growth	260 (43)	322 (54)	191 (32)	221 (37)					
Confluent multinodular or infiltrative type	9 (1)	11 (2)	3 (0.5)	33 (6)					
Imaging features associated with tumor burden									
Tumor number									
Solitary	530 (88)	522 (87)	521 (87)	524 (87)	567 (95)	589 (98)	588 (98)	0.696 (0.518–0.873)	0.901 (0.884–0.916)
2–3 tumors	61 (10)	70 (12)	69 (12)	61 (10)					
Over 3 tumors	9 (2)	8 (1)	10 (2)	15 (3)					
Tumor number (solitary vs. multiple)	70 (12)	78 (13)	79 (13)	76 (13)	567 (95)	589 (98)	588 (98)	0.696 (0.518–0.873)	0.689 (0.643–0.736)
Satellite tumors (present vs. absent)	26 (4)	29 (5)	37 (6)	44 (7)	574 (96)	590 (98)	583 (97)	0.659 (0.298–1.000)	0.458 (0.412–0.504)
Imaging features associated with the severity of underlying liver diseases and portal hypertension									
Ascites (present vs. absent)	22 (4)	28 (5)	29 (5)	46 (8)	587 (98)	589 (98)	593 (99)	0.557 (0.312–0.802)	0.331 (0.284–0.377)
Radiologically-evident cirrhosis (present vs. absent)	170 (28)	193 (32)	81 (14)	213 (36)	489 (82)	531 (89)	535 (89)	0.630 (0.518–0.742)	0.384 (0.337–0.430)
Diffuse iron overload (present vs. absent)	67 (11)	124 (21)	35 (6)	74 (12)	537 (90)	582 (97)	569 (95)	0.412 (0.253–0.586)	0.399 (0.352–0.445)
Diffuse fatty change (present vs. absent)	60 (10)	65 (11)	60 (10)	68 (11)	567 (95)	577 (96)	588 (98)	0.664 (0.507–0.822)	0.634 (0.588–0.681)
Width of main portal vein (cm)	…	…	…	…	589 (99.6)	598 (99.6)	599 (99.8)	0.917 (0.891–0.937)	0.686 (0.646–0.722)
Splenomegaly (present vs. absent)	338 (56)	323 (54)	431 (72)	266 (44)	517 (86)	541 (90)	560 (93)	0.683 (0.583–0.784)	0.448 (0.402–0.494)
Collateral circulation (present vs. absent)	357 (60)	367 (61)	454 (76)	246 (41)	504 (84)	551 (92)	561 (94)	0.465 (0.338–0.592)	0.291 (0.245–0.337)
Esophageal gastric varices (present vs. absent)	248 (40)	314 (52)	231 (39)	190 (32)	447 (75)	530 (88)	545 (91)	0.457 (0.342–0.572)	0.335 (0.289–0.382)

Unless stated otherwise, data in parentheses are 95% confidence intervals or percentages. R1, R2, and R3 were reviewers with seven, three, and ten years of experiences in liver MRI, respectively

^*^ Intra- and inter-observer agreements on continuous or ordinal/categorical variables were assessed with the intraclass correlation coefficient (ICC) or the weighted κ value, respectively. For binary variables, intra-observer agreements were evaluated with the Cohen's κ value, while the inter-observer agreements with the Fleiss' κ value. Agreement was considered poor (κ or ICC < 0.2), fair (κ or ICC: 0.2–0.4), moderate (κ or ICC: 0.4–0.6), substantial (κ or ICC: 0.6–0.8), or excellent (κ or ICC > 0.8)

^†^ Analyses were conducted for patients who underwent hepatobiliary contrast agent-enhanced MRI (*n* = 183)

*LI-RADS* = Liver Imaging Reporting and Data System; *TP* = transitional phase; *HBP* = hepatobiliary phase

### Clinical-radiological-pathological correlations of prognostic imaging markers

Larger tumor size was associated with more frequent MVI (Spearman’s *rho* = 0.41, *p* < 0.001) and the MTM subtype (Spearman’s *rho* = 0.24, *p* < 0.001). Tumor multiplicity was associated with increased MVI (57% vs. 32%, *p* = 0.003) and positive CK19 expression (32% vs. 13%, *p* = 0.02). Nonperipheral washout was associated with more frequent poor tumor differentiation (35% vs. 19%, *p* < 0.001), increased MVI (38% vs. 23%, *p* = 0.01), more frequent MTM subtype (28% vs. 8% *p* = 0.03), and less frequent positive CK19 expression (13% vs. 28%, *p* = 0.048). Rim APHE was associated with more frequent positive CK19 expression (32% vs. 13%, *p* = 0.04). Intratumoral arteries were associated with more frequent poor tumor differentiation (42% vs. 27%, *p* < 0.001), MVI (64% vs. 23%, *p* < 0.001), and MTM subtype (31% vs. 13%, *p* = 0.003). The VICT2 trait was associated with serum AFP > 400 ng/mL (33% vs. 23% *p* = 0.03), more frequent poor tumor differentiation (41% vs. 28%, *p* = 0.004), MVI (58% vs. 8%, *p* < 0.001), and MTM subtype (32% vs. 13%, *p* = 0.003). Serum AFP > 400 ng/mL (*p* = 0.004), MVI (*p* < 0.001), and positive CK19 expression (*p* = 0.005) were increasingly observed in patients with single nodular, single nodular with extranodular growth, and confluent multinodular or infiltrative subtypes. The presence of gsatroesophageal varices was correlated with the need for intraoperative transfusion (9% vs. 2%, *p* < 0.001).

**Fig. 5:**
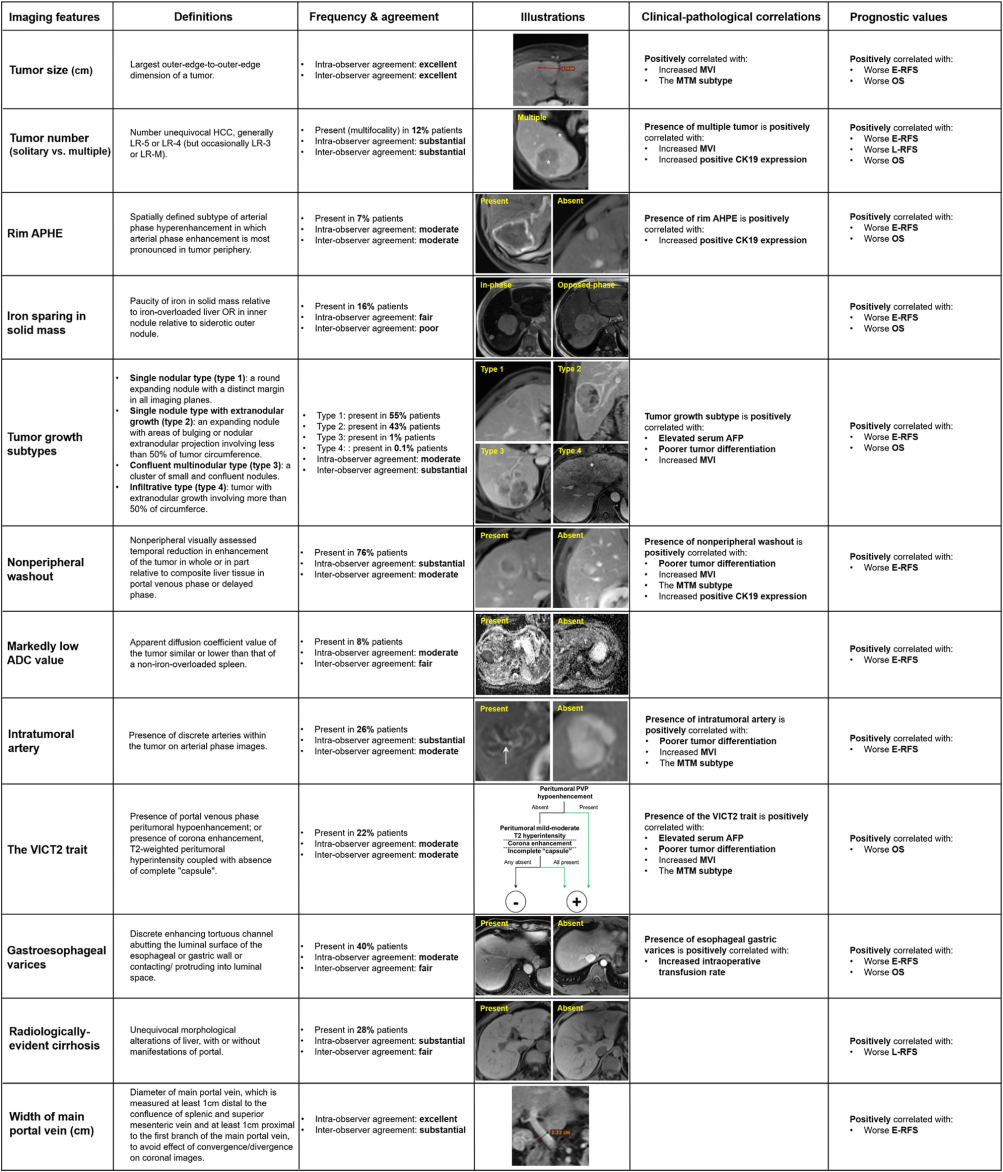
Definitions, frequencies, agreement, illustrations, clinical-pathological correlations, and prognostic utilities of the imaging markers. The VICT2 trait is considered positive when peritumoral PVP hypoenhancement is present or if corona enhancement, peritumoral mild-moderate T2 hypointensity, and incomplete capsule were all present; otherwise, negative. Abbreviations: APHE, arterial phase hyperenhancement; HCC, hepatocellular carcinoma; MVI, microvascular invasion; MTM, macrotrabecular-massive; CK19, cytokeratin 19; AFP, α-fetoprotein; E-RFS, early recurrence-free survival (i.e., recurrence-free survival within 2 years after surgery); L-RFS, late recurrence-free survival (i.e., recurrence-free survival beyond 2 years after surgery); OS, overall survival

The clinical-radiological-pathological correlations of the tumor-related prognostic imaging features are summarized in Table 5. Definitions, illustrations, and clinical implications of the prognostic imaging features are summarized in Fig. 5.

**Table 5:** Clinical-radiological-pathological correlations of the tumor-based prognostic imaging markers

Imaging features	Serum AFP		Differentiation		Microvascular invasion		The MTM subtype		CK19 expression	
	> 400 ng/mL	≤ 400 ng/mL	Poor	Well-moderate	Present	Absent	Positive	Negative	Positive	Negative
	(*n* = 150)	(*n* = 446)	(*n* = 181)	(*n* = 404)	(*n* = 104)	(*n* = 196)	(*n* = 36)	(*n* = 159)	(*n* = 27)	(*n* = 148)
Tumor number										
Multiple	20	49	21	47	20	15	3	154	7	15
Solitary	130	397	160	357	84	181	33	5	20	133
* p value*	*0.44*		*0.99*		***0.003***		*0.16*		***0.02***	
Nonperipheral washout										
Present	120	330	154	289	88	141	32	114	19	127
Absent	30	116	27	115	16	55	4	45	8	21
* p value*	*0.14*		** < *.001***		***0.01***		***0.03***		***0.048***	
Iron sparing in solid mass										
Present	22	70	27	65	21	33	7	26	3	27
Absent	128	376	154	339	83	163	29	133	24	121
* p value*	*0.76*		*0.72*		*0.47*		*0.66*		*0.37*	
Rim arterial phase hyperenhancement										
Present	10	29	14	23	6	14	1	11	6	13
Absent	140	417	167	381	98	182	36	148	21	135
* P value*	*0.94*		*0.35*		*0.65*		*0.35*		***0.04***	
Markedly low apparent diffusion coefficient value										
Present	11	34	15	375	12	13	2	9	4	9
Absent	139	412	166	29	92	183	34	150	23	139
* p value*	*0.91*		*0.64*		*0.08*		*0.98*		*0.11*	
Intratumoral artery										
Present	46	109	64	87	56	32	18	40	7	36
Absent	104	337	117	317	48	164	18	119	20	112
* p value*	*0.13*		** < *.001***		** < *.001***		***0.003***		*0.86*	
The VICT2 trait										
Present	42	87	53	75	41	30	18	39	10	31
Absent	108	359	128	329	63	166	18	120	17	117
* p value*	***0.03***		***0.004***		** < *.001***		***0.003***		*0.07*	
Tumor growth subtype										
Single nodular type	69	260	95	229	38	117	15	85	8	83
Single nodule type with extranodular growth	76	182	81	171	59	79	20	72	18	65
Confluent multinodular or infiltrative type	5	4	5	4	7	0	1	2	1	0
* p value*	***0.003***		*0.21*		** < *.001***		*0.17*		***0.005***	

Data are shown for patients who had complete documentations of these factors. All *p *values < .05 are highlighted in bold

*AFP* = α-fetoprotein; *MTM* = macrotrabecular-massive; *CK19* = cytokeratin 19

## Discussion

Individualized prognostication is paramount for improving HCC survival. Based on 600 patients who received curative-intent liver resection for BCLC 0-B stage HCCs, we identified 12 easily accessible MR imaging features that were predictive of postoperative E-RFS (≤ 2 years), L-RFS (> 2 years), and OS, independently from the majority of established prognostic factors.

In a well-characterized surgical cohort, we explored the prognostic values of 54 readily measurable imaging features. The prognostic values of most features were retained in subgroup analyses, highlighting their incremental values over existing prognostic factors. Among these features, eight tumor-related (e.g., tumor number, size, enhancement, and growth patterns) and two portal hypertension-related features (i.e., the width of the main portal vein and gastroesophageal varices) were associated with early recurrence, which accounts for approximately 70% of all recurrence events [1]. These features may help detect those high-risk patients who could potentially benefit from neoadjuvant and/or adjuvant therapies and are likely candidates for clinical trials [22]. These patients may also benefit from more sensitive postoperative surveillance approaches (e.g., contrast-enhanced MRI over CT or ultrasound).

Only radiologically evident cirrhosis and tumor multiplicity were associated with worse L-RFS. These results were in line with previous studies, which supports the hypothesis that while early recurrence is more linked to the tumor’s characteristics and may originate from the intrahepatic metastatic tumor foci, late recurrence is likely more associated with de novo tumors from the underlying chronic liver diseases [22, 23]. Our findings implied that patients with multiple tumors and/or radiologically evident cirrhosis may benefit from continued intensive surveillance beyond two years after liver resection.

Seven imaging features were independent risk factors for OS. Several of these features (e.g., tumor size, number, rim APHE) have been previously correlated with OS in HCC [25–27]. Interestingly, six of them (86%) were also correlated with E-RFS. These findings underscored the potential of preoperative MRI features in profiling the intrinsic and constant biological behaviors of tumors and implied that tumor burden and aggressiveness may be the predominant determinants for long-term survival.

The clinical-radiological-pathological correlations need to be understood before integrating these prognostic imaging markers in treatment decision-making. Specifically, rim APHE has been previously correlated with increased liver stem cell-like traits (e.g., increased CK19 expression), the proliferative subtype, increased MVI, and a more hypoxic and fibrotic tumor microenvironment [27–30]. We observed a similar correlation between rim APHE and increased CK19 expression. Furthermore, the tumor growth subtype, an imaging analogue of the pathologic gross subtype, represented a trend toward increasing tumor aggressiveness [31]. Alongside this trend, poorer differentiation, reduced capsule formation, increased MVI, and higher expression of stemness-related markers have been observed previously [32]. Similarly, more aggressive tumor growth subtypes were also associated with elevated serum AFP levels and increased MVI and CK19 expressions in our work. Iron sparing in solid mass was another adverse prognostic marker in this work. This feature has been previously described as a diagnostic feature for HCC [33]. The rationale for why this feature may portend a worse prognosis may be explained by the synergistic effect of liver iron accumulation (leading to increased oxidative stress) and relative tumor iron resistance (likely owing to *HAMP*/hepcidin downregulation, CDK-1/STAT3 pathway activation, and transferrin receptor-1 upregulation) [34–37].

Nonperipheral washout, specifically associated with worse E-RFS in this work, has been previously linked to relative portal tract reduction at pathology [38]. Our work further revealed a positive correlation between nonperipheral washout and poorer tumor differentiation. Intratumoral arteries were also specifically associated with early recurrence in our work, and it has been previously correlated with increased tumor angiogenesis and elevated risk of MVI [9, 11]. We confirmed its correlation with MVI while also finding a positive correlation with poorer tumor differentiation. Additionally, the presence of the VICT2 trait, a recently reported non-hepatobiliary-specific analogue of peritumoral HBP hypointensity [12], was specifically associated with worse OS in the current work. This feature was also associated with elevated serum AFP, poorer tumor differentiation, increased MVI, and worse OS in our work.

Noteworthily, instead of directly developing prognostic models, the current work systemically investigated the prognostic values, reliability, and clinical-radiological-pathological correlations for a total of 54 MRI features in a large, uniform, and well-controlled patient cohort with relatively long follow-up period, because we think effective and generalizable prognostic models which may alter the therapeutic workflow should only include robust, reproducible, and explainable imaging features. Therefore, the identifications of prognostic MRI features for different survival outcomes may be integrated into future prognostic models. However, most of them were highly dependent on the radiologists’ experiences with unsatisfactory intra- and inter-observer agreement. Therefore, efforts are required to enhance the repeatability and reproducibility of these subjectively interpreted findings, perhaps through more streamlined terminology, standardized training, and artificial intelligence techniques [39].

This study has several limitations. First, as a single-center study, no external validation was available to test our findings. Second, up to 95% of our enrolled patients had chronic hepatitis B, which may limit the extrapolations of our findings in the non-HBV population. Third, the postoperative surveillance interval varied between three to six months. While these intervals were determined according to the practice guidelines [3], these variations may have negatively impacted the detection of recurrence. Fourth, due to the study’s retrospective nature and that the pathological data were retrieved from routine reports, a substantial number of patients had inadequate documentation on the clinical and pathological characteristics, which may have introduced selection biases and influenced the statistical power of the subgroup analyses as well as the assessments of clinical-radiological-pathological correlations. Finally, the clinical implications and biological underpinnings of these prognostic imaging markers were largely hypothetical, with low levels of evidence. Therefore, further larger-scale multi-center studies enrolling patients with different chronic liver disease etiologies are warranted to validate and extrapolate our findings.

In summary, based on 600 patients who received curative-intent liver resection for BCLC 0-B stage HCCs, we identified 12 easily measurable MRI features that were predictive of postoperative E-RFS (≤ 2 years), L-RFS (> 2 years), and OS, regardless of the majority of known prognostic factors. These prognostic features may help inform personalized surgical planning, neoadjuvant and/or adjuvant therapies, and tailor postoperative surveillance strategies, thus may be integrated into future prognostic models.

### Supplementary Information

Below is the link to the electronic supplementary material.

Supplementary file1 (PDF 741 KB)

## Abbreviations

AFP: α-Fetoprotein
APHE: Arterial phase hyperenhancement.
BCLC: Barcelona Clinic Liver Cancer
CK19: Cytokeratin 19
E-RFS: Early-recurrence-free survival
HBP: Hepatobiliary phase
HCC: Hepatocellular carcinoma
ICC: Intraclass correlation coefficient
LI-RADS: Liver Imaging Reporting and Data System
L-RFS: Late-recurrence-free survival
MTM: Macrotrabecular-massive
MVI: Microvascular invasion
OS: Overall survival
RFS: Recurrence-free survival

## Declarations

### Guarantor

The scientific guarantor of this publication is Hanyu Jiang.

### Statistics and biometry

Two of the authors (Yuanan Wu and Chengyu Ding) have significant statistical expertise.

### Study subjects or cohorts overlap

262 patients included in the current study have been reported previously [1]. While the prior work proposed a non-hepatobiliary-specific MR alternative to HBP peritumoral hypointensity, the current work investigated the prognostic values, reliability, and clinical-radiological-pathological correlations for currently available MRI prognostic features.

1. Jiang H, Wei H, Yang T, et al (2023) VICT2 trait: prognostic alternative to peritumoral HBP hypointensity in hepatocellular carcinoma. Radiology. 307(2):e221835. 10.1148/radiol.221835

### Methodology

• Retrospective

• diagnostic or prognostic study

• performed at one institution
