# Supplementary material for: Prognostic MRI features to predict postresection survivals for very early to intermediate stage hepatocellular carcinoma
Source: Eur Radiol. 2023 Oct 23;34(5):3163–82. doi: 10.1007/s00330-023-10279-x (PMC11126450; doi:10.1007/s00330-023-10279-x)
Supplement: Supplementary file 1 — Supplementary file1 (PDF 741 KB) [file 330_2023_10279_MOESM1_ESM.pdf]

Prognostic MRI features to predict postresection survivals for very early to intermediate stage hepatocellular carcinoma

Electronic Supplementary Material

Table of contents

Supplemental Material 1: Imaging acquisition protocols 2

Supplemental Table 2: Definitions of the imaging predictors......................................8

Supplemental Figures 14

Supplemental Material 1: Imaging acquisition protocols

All contrast-enhanced MR images were acquired on one of the following 3.0 T or 1.5 T MR systems (Siemens MAGNETOM Skyra 3.0 T; Siemens TrioTim 3.0 T; Siemens Avanto 1.5 T; GE SIGNA™ Architect 3.0 T; GE SIGNA™ Premier 3.0 T; GE Discovery MR 750 3.0 T; Philips Ingenia Elition X 3.0 T; uMR588 1.5 T) using either extracellular contrast-enhanced MRI (ECA-MRI) or gadoxetate disodium-enhanced MRI (EOB-MRI). For acquisition of ECA-MRI, 0.1 mmol/kg of gadopentetate dimeglumine (Magnevist®; Bayer Schering Pharma AG), gadoterate meglumine (Dotarem®; Guerbet), or gadobenate dimeglumine (MultiHance®; Bracco) was injected at a rate of 2.5 ml/s. For acquisition of EOB-MRI, 0.025 mmol/kg of EOB (Primovist®; Bayer Schering Pharma AG or XianAi®; Chia Tai TianQing Pharmaceutical Co., Ltd) was injected at a rate of 1.0-2.0 ml/s.

All patients were asked to fast for 6-8 hours before MR examinations. The MRI sequences included: (a) T2-weighted imaging; (b) diffusion-weighted imaging (b values: 0, 50, 500, 800, 1000, and 1200 s/mm2 [Siemens MAGNETOM Skyra]; 0, 50, 800 s/mm2 [Siemens TrioTim; Siemens Avanto]; 0, 50, 1000 s/mm2 [Siemens Avanto; GE SIGNA™ Architect; GE SIGNA™ Premier; uMR588]; 0, 200, 800, and 1000 s/mm2 [GE Discovery MR 750]; 0, 200, 1000 s/mm2 [Philips Ingenia Elition X]) with apparent diffusion coefficient maps reconstructed using the monoexponential model; (c) in- and opposed-phase T1-weighted imaging; and (d) dynamic T1-weighted imaging in the pre-contrast phase, late arterial phase, portal venous phase (60 s after the start of contrast media injection), delayed phase (for ECA-MRI, 3min after start of contrast media injection) or transitional phase (for EOB-MRI, 5min after the start of contrast media injection), and hepatobiliary phase (for EOB-MRI, 20min after the start of contrast media injection). The arterial phase images were acquired with either a bolus-tracking method (acquisition triggered 7s after the arrival of the contrast bolus in the celiac trunk) or a multiple arterial phase imaging technique (acquired with an 18 s-breath-hold 20s after the start of contrast media injection and further reconstructed with a temporal resolution of 3 s).

MR acquisition parameters are detailed in **Supplemental Table 1**.

Supplemental Table 1. MR Sequences and Parameters

Sequence	Fat suppression	TR (ms)	TE (ms)	Flip angle (°)	ST (mm)	Spacing (mm)	Matrix size	FOV (mm^2)	Acquisition Time (s)
**Siemens MAGNETOM Skyra 3.0 Tesla (18-channel body array coil)**									
T2-weighted 2D FSE	Yes	2160	100	160	6	1.8	320×288	433×433	36
Diffusion-weighted imaging*	Yes	5600	68	90	6	1.8	100×76	380×289	233
In- and opposed-phase T1-weighted imaging	No	81	2.72/1.4	70	6	1.8	352×286	400×325	24
Dynamic T1-weighted 3D GRE	Yes	3.95	1.92	9	2.5	-	352×256	400×296	14
**Siemens TrioTim 3.0 Tesla (8-channel body anterior coil)**									
T2-weighted 2D FSE	Yes	2700	95	140	6	7.8	320×147	442×254	Respiratory gating
Diffusion-weighted imaging*	Yes	5900	76	90	6	7.8	192×154	393×393	245
In- and opposed-phase T1-weighted imaging	No	181	2.2/1.1	65	6	7.8	256×131	410×269	18
Dynamic T1-weighted 3D GRE	Yes	3.47	1.25	9	2.4	-	320×133	434×257	17

Note. —TR = repetition time; TE = echo time; ST = section thickness; FOV = field of view; 2D = two-dimensional; 3D = three-dimensional; FSE = fast spin-echo; GRE = gradient recall echo. * Images were acquired under free breath.

Sequence	Fat suppression	TR (ms)	TE (ms)	Flip angle (°)	ST (mm)	Spacing (mm)	Matrix size	FOV (mm^2)	Acquisition Time (s)
**Siemens Avanto 1.5 Tesla (30-channel body anterior coil)**									
T2-weighted 2D FSE	Yes	2530	84	150	6	7.8	256×187	293×251	47
Diffusion-weighted imaging*	Yes	3600	88	90	6	7.8	192×115	310×232	92
In- and opposed-phase T1-weighted imaging	No	72	4.92/2.22	70	6	7.8	256×158	328×225	16
Dynamic T1-weighted 3D GRE	Yes	5.41	2.39	10	2.5	-	320×138	382×238	15
**Siemens Avanto 1.5 Tesla (8-channel body anterior coil)**									
T2-weighted 2D FSE	Yes	2710	84	150	7.5	9.75	256×177	308×380	27
Diffusion-weighted imaging*	Yes	2000	72	90	7.5	9.75	192×125	308×379	20
In- and opposed-phase T1-weighted imaging	No	87	4.92/2.22	70	7.5	9.75	256×187	308×380	33
Dynamic T1-weighted 3D GRE	Yes	5.4	2.38	10	2	-	320×131	241×407	15

Note. —TR = repetition time; TE = echo time; ST = section thickness; FOV = field of view; 2D = two-dimensional; 3D = three-dimensional; FSE = fast spin-echo; GRE = gradient recall echo. * Images were acquired under free breath.

Sequence	Fat suppression	TR (ms)	TE (ms)	Flip angle (°)	ST (mm)	Spacing (mm)	Matrix size	FOV (mm^2)	Acquisition Time (s)
**GE SIGNA™ Architect 3.0 Tesla (30-channel body anterior coil)**									
T2-weighted 2D FSE	Yes	2400	85	111	7	2	320×192	380×304	34
Diffusion-weighted imaging*	Yes	5000	Minimum	90	7	2	160×128	380×342	Respiratory gating
In- and opposed-phase T1-weighted imaging	No	233.8	2.3/1.1	55	7	2	160×288	380×323	18
Dynamic T1-weighted 3D GRE	Yes	3.9	1.7	15	3	-	320×240	380×380	15
**GE SIGNA™ Premier 3.0 Tesla (30-channel body anterior coil)**									
T2-weighted 2D FSE	Yes	2200	85	111	7	2	320×224	304×380	47
Diffusion-weighted imaging*	Yes	5000	Minimum	90	7	2	120 × 240	380× 380	Respiratory gating
In- and opposed-phase T1-weighted imaging	No	146.8	2.3/1.1	55	7	2	320×192	342×380	16
Dynamic T1-weighted 3D GRE	Yes	3.2	1.4	15	2.4	-	320×240	380× 380	15

Note. —TR = repetition time; TE = echo time; ST = section thickness; FOV = field of view; 2D = two-dimensional; 3D = three-dimensional; FSE = fast spin-echo; GRE = gradient recall echo. * Images were acquired under free breath.

Sequence	Fat suppression	TR (ms)	TE (ms)	Flip angle (°)	ST (mm)	Spacing (mm)	Matrix size	FOV (mm^2)	Acquisition Time (s)
**GE Discovery MR 750 3.0 Tesla (16-channel phased-array torsor coil)**									
T2-weighted 2D FSE	Yes	6315	78	111	6	2	288×244	360×280	Respiratory gating
Diffusion-weighted imaging*	Yes	9230	Minimum	90	6	2	128 × 128	360× 380	Respiratory gating
In- and opposed-phase T1-weighted imaging	No	150	2.5/1.3	70	6	2	288×192	420×420	31
Dynamic T1-weighted 3D GRE	Yes	4.1	1.9	15	2	-	512×512	380× 300	15
**Philips Ingenia Elition X 3.0 Tesla (16-channel body anterior coil)**									
T2-weighted 2D FSE	Yes	1883.51	90	90	6.8	8.5	272×78	239×69	46
Diffusion-weighted imaging*	Yes	1653.65	60.29	90	7	8.5	142×140	141×139	52
In- and opposed-phase T1-weighted imaging	No	164.53	2.30/1.15	50	6	7.5	256×201	206×162	11
Dynamic T1-weighted 3D GRE	Yes	4.20	0.00	10	3	1.5	344×252	303×222	13

Note. —TR = repetition time; TE = echo time; ST = section thickness; FOV = field of view; 2D = two-dimensional; 3D = three-dimensional; FSE = fast spin-echo; GRE = gradient recall echo. * Images were acquired under free breath.

Sequence	Fat suppression	TR (ms)	TE (ms)	Flip angle (°)	ST (mm)	Spacing (mm)	Matrix size	FOV (mm^2)	Acquisition Time (s)
**uMR588 1.5 Tesla (6-channel body anterior coil)**									
T2-weighted 2D FSE	Yes	2600	99.2	90	6.5	1.5	256×168	427×320	39
Diffusion-weighted imaging*	Yes	3350	77	90	6.5	10	128×92	320×400	Respiratory gating
In- and opposed-phase T1-weighted imaging	No	117.6	4.7/2.27	60	6.5	1.3	256×174	320×400	29
Dynamic T1-weighted 3D GRE	Yes	4.2	1.88	10	2.5	-	256×154	255×400	13

Note. —TR = repetition time; TE = echo time; ST = section thickness; FOV = field of view; 2D = two-dimensional; 3D = three-dimensional; FSE = fast spin-echo; GRE = gradient recall echo. * Images were acquired under free breath.

Supplemental Table 2: Definitions of the imaging predictors

Imaging features	Definition	Image sequence
LI-RADS major features		
Nonrim arterial phase hyperenhancement (present vs. absent)	Nonrim-like enhancement of the tumor in the arterial phase unequivocally greater in whole or in part than the liver. (1,2)	Late arterial phase
Nonperipheral washout (present vs. absent)	Nonperipheral visually assessed temporal reduction in the enhancement of the tumor in whole or in part relative to composite liver tissue in the portal venous phase or delayed phase. (1,2)	Portal venous or delayed phase for extracellular contrast agents or gadobenate; portal venous phase for gadoxetate
Enhancing capsule (present vs. absent)	Smooth, uniform, sharp border around most or all of a tumor, unequivocally thicker or more conspicuous than fibrotic tissue around background nodules, and visible as enhancing rim in portal venous phase, delayed phase, or transitional phase. (1,2)	Portal venous, delayed, or transitional phase
Tumor size (cm)	Largest outer-edge-to-outer-edge dimension of a tumor. (1,2)	Pick the phase, sequence, plane in which the margins are clearest. Do not measure in arterial phase or DWI.
LI-RADS ancillary features		
Diffusion restriction (present vs. absent)	Signal intensity of the tumor higher than the liver on diffusion-weighted images not caused only by T2 shine-through. (1,2)	High b value (e.g.,≥800) DWI
Mild-moderate T2 hyperintensity (present vs. absent)	Signal intensity of the tumor on T2WI higher than the liver, similar to or lower than a non-iron-overloaded spleen, and lower than simple fluid. (1,2)	T2WI
Corona enhancement (present vs. absent)	Peritumoral enhancement in the late arterial phase or early portal venous phase. The enhancement is contiguous with and surrounds all or part of the tumor. (1,2)	Late arterial or early portal venous phase
Nonenhancing capsule (present vs. absent)	Subtype of capsule that does not show enhancement on any image. (1,2)	Multiple sequences
Nodule-in-nodule architecture (present vs. absent)	Presence of smaller inner nodule within and having different imaging features than larger outer nodule. (1,2)	Multiple sequences
Mosaic architecture (present vs. absent)	Presence of any combination of internal nodules, compartments, or septations, within a solid or mostly solid mass. (1,2)	Multiple sequences
Blood products in mass (present vs. absent)	Intralesional or perilesional hemorrhage in the absence of biopsy, trauma, or intervention. (1,2)	Multiple sequences
Fat in mass, more than adjacent liver (present vs. absent)	Excess fat within a mass, in whole or in part, relative to the adjacent liver. (1,2)	In-/opposed phase; fat-suppressed images compared to non-fat-suppressed images with similar or identical weighting

Fat sparing in solid mass (present vs. absent)	Relative paucity of fat in solid mass relative to steatotic liver OR in inner nodule relative to steatotic outer nodule. (1,2)	In-/opposed phase; fat-suppressed images compared to non-fat-suppressed images with similar or identical weighting
Iron sparing in solid mass (present vs. absent)	Paucity of iron in solid mass relative to iron-overloaded liver OR in inner nodule relative to siderotic outer nodule. (1,2)	In-/opposed phase; T2WI
Transitional phase hypointensity (preset vs. absent)	Signal intensity of the tumor in the transitional phase unequivocally less, in whole or in part, than the liver. (1,2)	Transitional phase
Hepatobiliary phase hypointensity (preset vs. absent)	Signal intensity of the tumor in the hepatobiliary phase unequivocally less, in whole or in part, than the liver. (1,2)	Hepatobiliary phase
Marked T2 hyperintensity (present vs. absent)	Signal intensity of the tumor on T2WI higher than non-iron-overloaded spleen and as high as or almost as high as simple fluid. (1,2)	T2WI
Iron in mass, more than liver (present vs. absent)	Excess iron in a mass relative to the background liver. (1,2)	In-/opposed phase; T2WI
Parallels blood pool enhancement (present vs. absent)	Temporal pattern in which enhancement approximates blood pool in all phases. (1,2)	Post-contrast phases
Undistorted vessels (present vs. absent)	Vessels traversing a tumor without displacement, deformation, or other alteration. (1,2)	Post-contrast phases
LR-M features		
Rim arterial phase hyperenhancement (present vs. absent)	Spatially defined subtype of arterial phase hyperenhancement in which arterial phase enhancement is most pronounced in tumor periphery. (1,2)	Late arterial phase
Peripheral washout (present vs. absent)	Presence of apparent washout most pronounced in the tumor periphery. (1,2)	Portal venous or delayed phase for extracellular contrast agents or gadobenate; portal venous phase for gadoxetate
Delayed central enhancement (present vs. absent)	Central area of progressive postarterial phase enhancement. (1,2)	Postarterial phases
Targetoid restriction (present vs. absent)	Concentric pattern on diffusion-weighted imaging characterized by restricted diffusion in tumor periphery with less restricted diffusion in tumor center. (1,2)	DWI or ADC map
Targetoid transitional phase or hepatobiliary phase appearance (present vs. absent)	Concentric pattern in transitional or hepatobiliary phase characterized by moderate-to-marked hypointensity in the tumor periphery with milder hypointensity in the center. (1,2)	Transitional or hepatobiliary phase
Marked diffusion restriction (present vs. absent)	Signal intensity of the tumor similar to or higher than non-iron-overloaded spleen on diffusion-weighted images not caused only by T2 shine-through. (1,2)	High b value (e.g.,≥800) DWI

Infiltrative appearance (present vs. absent)	Presence of a non-circumscribed tumor margin (indistinct transition) thought to represent a permeative growth pattern. (1,2)	Multiple sequences
Necrosis or severe ischemia (present vs. absent)	Presence of nonenhancing area in a solid mass, not attributable to a cystic component, prior treatment, or intralesional hemorrhage. (1,2)	Post-contrast phases and T2WI
Other tumor-related prognostic features		
Pre-contrast T1-weighted hypointensity (present vs. absent)	Signal intensity of the tumor on pre-contrast T1-weighted imaging unequivocally lower than that of the liver.	Pre-contrast T1WI
T2-weighted peritumoral hyperintensity (present vs. absent)	Presence of irregular, wedge-shaped, or flame-like area adjacent to the tumor on T2WI of which the signal intensity is mildly or moderately higher than liver and similar to or less than a non-iron-overloaded spleen. (3,4)	T2WI
Portal venous phase peritumoral hypoenhancement (present vs. absent)	Presence of irregular, wedge-shaped, or flame-like hypoenhancing area adjacent to the tumor in the portal venous phase. (3,4)	Portal venous phase
Markedly low apparent diffusion coefficient value (present vs. absent)	Apparent diffusion coefficient value of the tumor similar or lower than that of a non-iron-overloaded spleen.	ADC map
≥50% arterial phase hyperenhancement (present vs. absent)	Presence of ≥50% tumor volume demonstrating arterial phase hyperenhancement. (5)	Late arterial phase
Intratumoral artery (present vs. absent)	Presence of discrete arteries within the tumor on arterial phase images. (3,6,7)	Early or late arterial phase
Complete "capsule" (present vs. absent)	Presence of non-disrupted “capsule” in all imaging planes. (3,8)	Multiple sequences
Non-smooth tumor margin (present vs. absent)	The tumor margin is irregular and/or has areas of bulging, nodular projection, or infiltration into adjacent tissues at the tumor periphery in any imaging plane. (3,7,10)	Multiple sequences
Marked hepatobiliary phase hypointensity (present vs. absent)	Signal intensity of the tumor in the hepatobiliary phase lower than that of the liver and similar to or lower than that of intrahepatic vessels. (11)	Hepatobiliary phase
Hepatobiliary phase peritumoral hypointensity (present vs. absent)	Presence of irregular, wedge-shaped or flame-like hypointense area of liver parenchyma located outside of the tumor margin in the hepatobiliary phase. (3,4,11,12)	Hepatobiliary phase
The VICT2 trait (present vs. absent)	Presence of portal venous phase peritumoral hypoenhancement; or presence of corona enhancement, T2-weighted peritumoral hyperintensity coupled with absence of complete "capsule". (4)	Multiple sequences
The TTPVI trait (present vs. absent)	Presence of intratumoral artery coupled with absence of nonenhancing "capsule". (6,7)	Multiple sequences

Tumor growth subtype (13,14)		Multiple sequences
Single nodular type	A round expanding nodule with a distinct margin in all imaging planes.	
Single nodule type with extranodular growth	An expanding nodule with areas of bulging or nodular extranodular projection involving less than 50% of tumor circumference.	
Confluent multinodular type*†	A cluster of small and confluent nodules.	
Infiltrative type†	Tumor with extranodular growth involving more than 50% of circumference.	
Imaging features associated with tumor burden		
Tumor number (solitary vs. 2-3 tumors vs. over 3 tumors/solitary vs. multiple)	Number of unequivocal HCC, generally LR-5 or LR-4 (but occasionally LR-3 or LR-M). (1,11,15-18)	Multiple sequences
Satellite tumors* (present vs. absent)	Presence of separate unequivocal HCCs within 2 cm of the border of the dominant tumor.	Multiple sequences
Imaging features associated with the severity of underlying liver diseases and portal hypertension		
Ascites (present vs. absent)	Presence of free fluid in abdomen or pelvis. (19)	T2WI
Radiologically-evident cirrhosis (present vs. absent)	Unequivocal morphological alterations of the liver, including surface nodularity, small liver volume, expansion of space between liver and anterior abdominal wall and perihilar, gallbladder fossa and ligamentum teres spaces, hypertrophy of caudate and/or left lateral section, atrophy of anterior right section and/or left medial section, anterolateral flattening, notching of posterior medial right lobe and parenchymal nodules, with or without manifestations of portal hypertension (portal-systemic collaterals, splenomegaly and/or ascites). (1,2)	Multiple sequences
Diffuse iron overload (present vs. absent)	Diffuse signal intensity drop of the liver parenchyma on in-phase images compared with the opposed-phase images. (1,2)	In-/opposed phase; T2WI
Diffuse fatty change (present vs. absent)	Diffuse signal intensity drop of the liver parenchyma on opposed-phase images compared with the in-phase images. (1,2)	In-/opposed phase; fat-suppressed images compared to non-fat-suppressed images with similar or identical weighting
Width of main portal vein (cm)	Diameter of main portal vein, which is measured at least 1cm distal to the confluence of splenic and superior mesenteric vein and at least 1cm proximal to the first branch of the main portal vein, to avoid effect of convergence/divergence on coronal images. (20)	Coronal images of the portal venous or delayed phase

Splenomegaly (present vs. absent)	Craniocaudal diameter of the spleen>12cm. (19,21,22)	Multiple sequences
Porto-systemic shunts (present vs. absent)	Enhancing tortuous channels in esophageal, epigastric, perisplenic, paraumbilical or retroperitoneal locations. (19)	Portal venous or delayed phase
Esophageal gastric varices (present vs. absent)	Discrete enhancing tortuous channel abutting the luminal surface of the esophageal or gastric wall or contacting/ protruding into luminal space. (19)	Portal venous or delayed phase

* The confluent multinodular subtype is different from satellite tumors as the former describes a cluster of small and confluent nodules, while the latter describes presence of separate unequivocal HCCs within 2 cm of the border of the dominant tumor.

† Tumor with the confluent multinodular or infiltrative type was considered solitary if no additional separate unequivocal HCC was identified, and the size measurement should encompass the entire confluent tumor cluster in the sequence/phase/plane that the margin is clearest.

References:

1. CT/MRI Liver Imaging Reporting and Data System version 2018. American College of Radiology Web site. https://www.acr.org/Clinical-Resources/Reporting-andData- Systems/LI-RADS/CTMRI-LI-RADS-v2018. Accessed 1 September 2021.
2. LI-RADS Lexicon (terms and definitions). American College of Radiology Web site. https://www.acr.org/-/media/ACR/Files/RADS/LI-RADS/LIRADS-Lexicon-Table.pdf. Accessed 1 September 2021.
3. Jiang H, Wei J, Fu F, et al. Predicting microvascular invasion in hepatocellular carcinoma: a dual-institution study on gadoxetate disodium-enhanced MRI. Liver Int. 2022;42(5):1158-1172.
4. Jiang H, Wei H, Yang T, et al. VICT2 trait: prognostic alternative to peritumoral HBP hypointensity in hepatocellular carcinoma. Radiology. 2023 Feb 14:221835.
5. Rhee H, Cho ES, Nahm JH, et al. Gadoxetic acid-enhanced MRI of macrotrabecular-massive hepatocellular carcinoma and its prognostic implications. J Hepatol. 2021;74(1):109-121.
6. Segal E, Sirlin CB, Ooi C, Adler AS, Gollub J, Chen X, et al. Decoding global gene expression programs in liver cancer by noninvasive imaging. Nat Biotechnol 2007; 25:675–680.
7. Renzulli M, Brocchi S, Cucchetti A, Mazzotti F, Mosconi C Sportoletti C, et al. Can Current Preoperative Imaging Be Used to Detect Microvascular Invasion of Hepatocellular Carcinoma? Radiology 2016;279(2):432-442.
8. Lei Z, Li J, Wu D, Xia Y, Wang Q, Si A, et al. Nomogram for Preoperative Estimation of Microvascular Invasion Risk in Hepatitis B Virus-Related Hepatocellular Carcinoma Within the Milan Criteria. JAMA 2016;151(4):356-363.
9. Lee S, Kim SH, Lee JE, Sinn DH, Preoperative gadoxetic acid-enhanced MRI for predicting microvascular invasion in patients with single hepatocellular carcinoma. J Hepatol 2017;67(3):526-534.
10. An C, Kim DW, Park YN, Chung YE, Rhee H, Kim MJ. Single hepatocellular carcinoma: preoperative MR imaging to predict early recurrence after curative resection. Radiology 2015; 276:433–443.
11. Fowler KJ, Chernyak V, Ronot M, et al. Hepatocellular Carcinoma: It Is Time to Focus on Prognosis. Radiology. 2023:220884.
12. Lee S, Kim SH, Lee JE, Sinn DH, Preoperative gadoxetic acid-enhanced MRI for predicting microvascular invasion in patients with single hepatocellular carcinoma. J Hepatol 2017;67(3):526-534.
13. Rhee H, Chung T, Yoo JE, et al. Gross type of hepatocellular carcinoma reflects the tumor hypoxia, fibrosis, and stemness-related marker expression. Hepatol Int. 2020;14(2):239-248.
14. Burt AD, Alves V, Bedossa P, et al. Data set for the reporting of intrahepatic cholangiocarcinoma, perihilar cholangiocarci noma and hepatocellular carcinoma: recommendations from the International Collaboration on Cancer Reporting (ICCR). Histopathology. 2018;73:369–85.
15. General Office of National Health Commission. Standard for diagnosis and treatment of primary liver cancer (2022 edition). J Clin Hepatol. 38 (2), 306-321.
16. European Association for the Study of the Liver; European Association for the Study of the Liver. EASL Clinical Practice Guidelines: Management of hepatocellular carcinoma. J Hepatol 2018;69(1):182-236.
17. Omata M, Cheng AL, Kokudo N, Kudo M, Lee JM, Jia J, et al. Asia-Pacific clinical practice guidelines on the management of hepatocellular carcinoma: a 2017 update. Hepatol Int 2017;11(4):317-370.

18. Marrero JA, Kulik LM, Sirlin CB, Zhu AX, Finn RS, Abecassis MM, et al. Diagnosis, Staging, and Management of Hepatocellular Carcinoma: 2018 Practice Guidance by the American Association for the Study of Liver Diseases. Hepatology. 2018;68(2):723-750.
19. Venkatesh SK, Yin M, Takahashi N, et al. Non-invasive detection of liver fibrosis: MR imaging features vs. MR elastography. Abdom Imaging. 2015;40(4):766-75.
20. Stamm ER, Meier JM, Pokharel SS, et al. Normal main portal vein diameter measured on CT is larger than the widely referenced upper limit of 13 mm. Abdom Radiol (NY). 2016;41(10):1931-6.
21. Rustogi R, Horowitz J, Harmath C, et al. Accuracy of MR elastography and anatomic MR imaging features in the diagnosis of severe hepatic fibrosis and cirrhosis. J Magn Reson Imaging. 2012;35(6):1356-64.
22. Marasco G, Colecchia A, Colli A, et al. Role of liver and spleen stiffness in predicting the recurrence of hepatocellular carcinoma after resection. J Hepatol. 2019;70(3):440-448.

Supplemental Figures

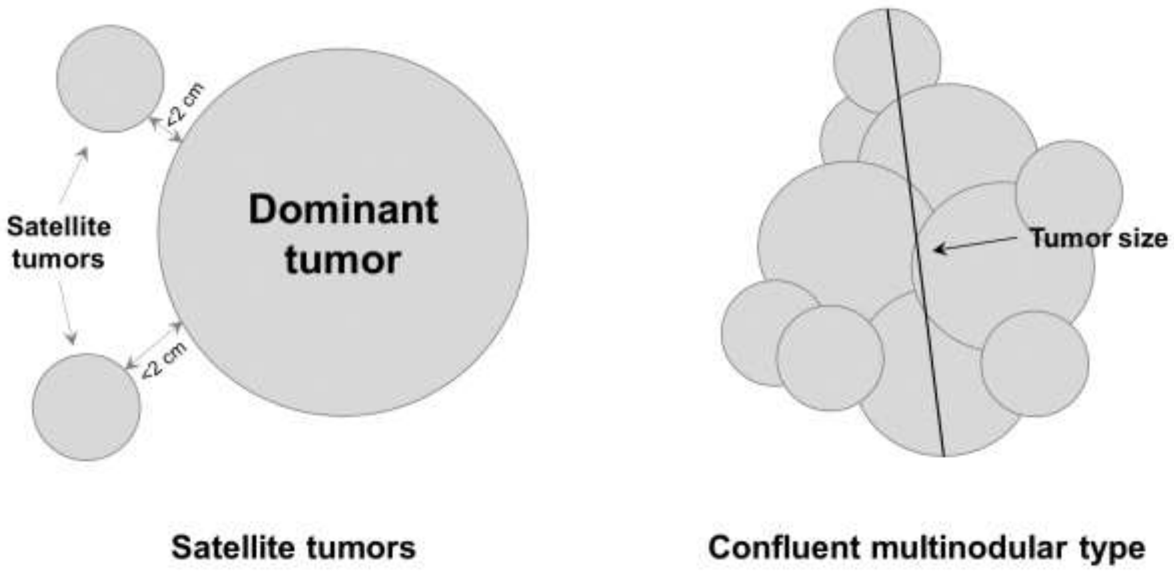

Supplemental Figure 1. Graphical comparisons of satellite tumors and the confluent multinodular type.

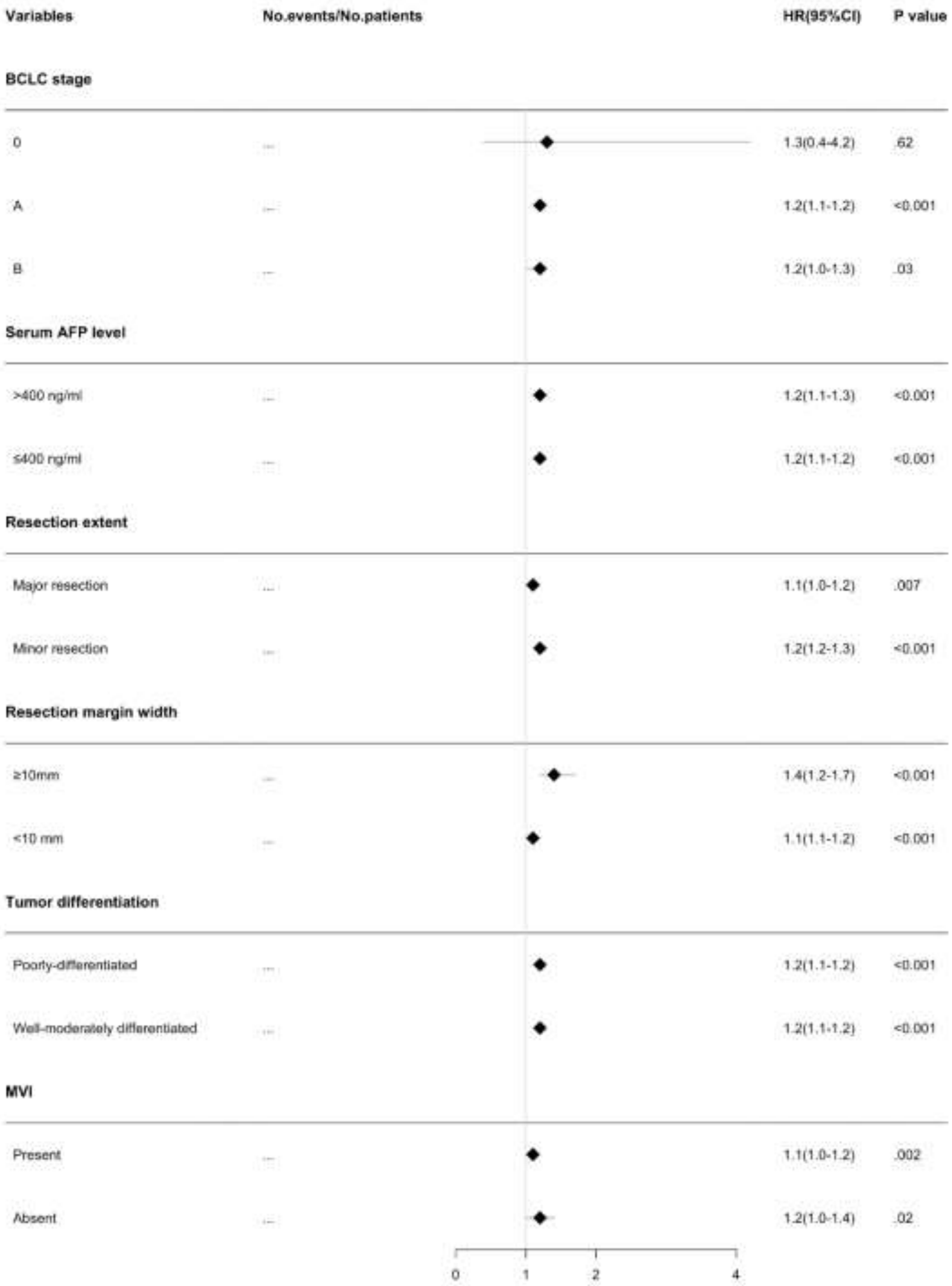

Supplemental Figure 2. Subgroup analyses for tumor size (cm) in predicting early recurrence-free survival based on six clinical-pathological prognostic factors.

BCLC, Barcelona Clinic Liver Cancer; AFP, α-fetoprotein; MVI, microvascular invasion.

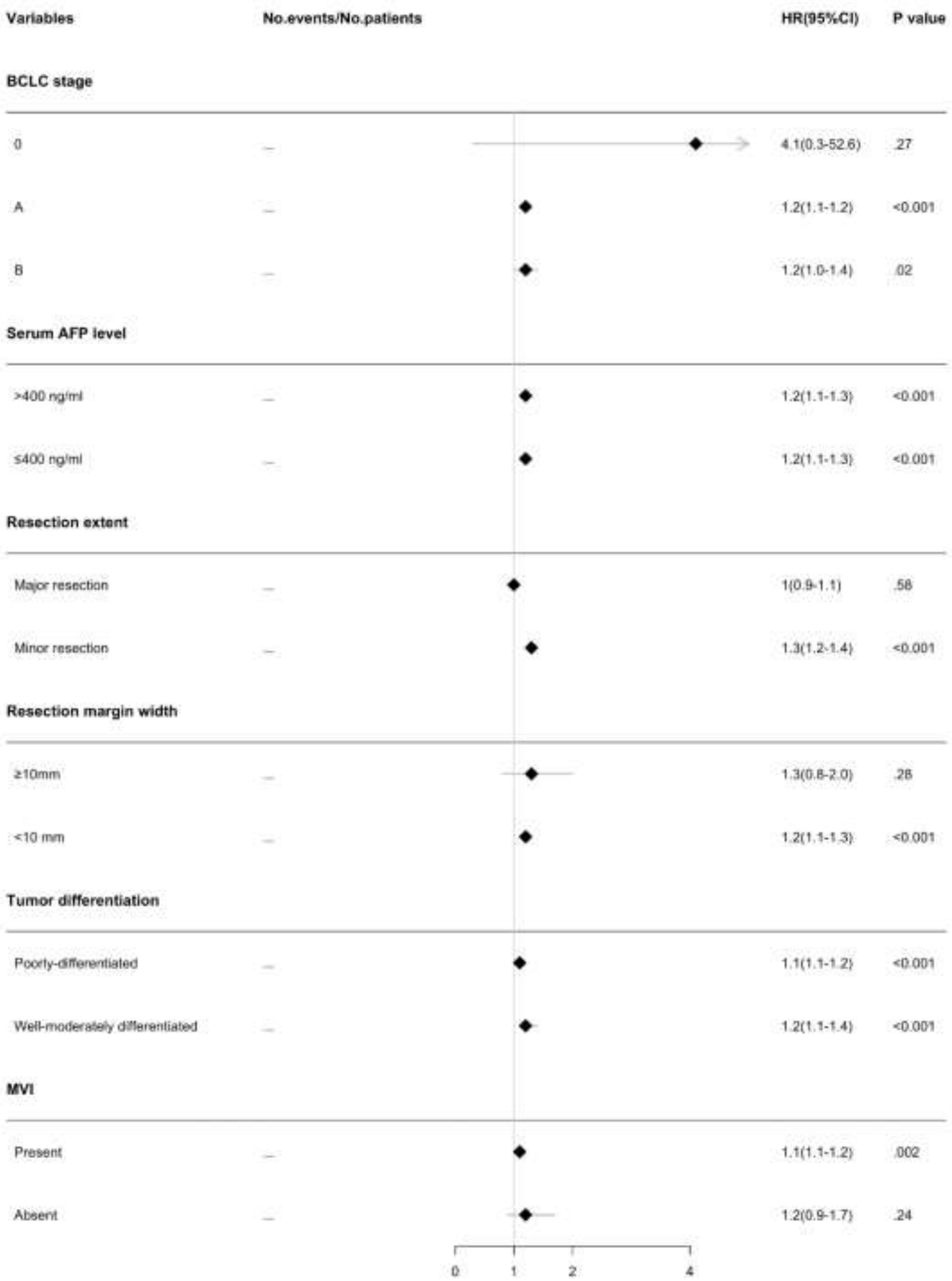

Supplemental Figure 3. Subgroup analyses for tumor size (cm) in predicting overall survival based on six clinical-pathological prognostic factors.

BCLC, Barcelona Clinic Liver Cancer; AFP, α-fetoprotein; MVI, microvascular invasion.

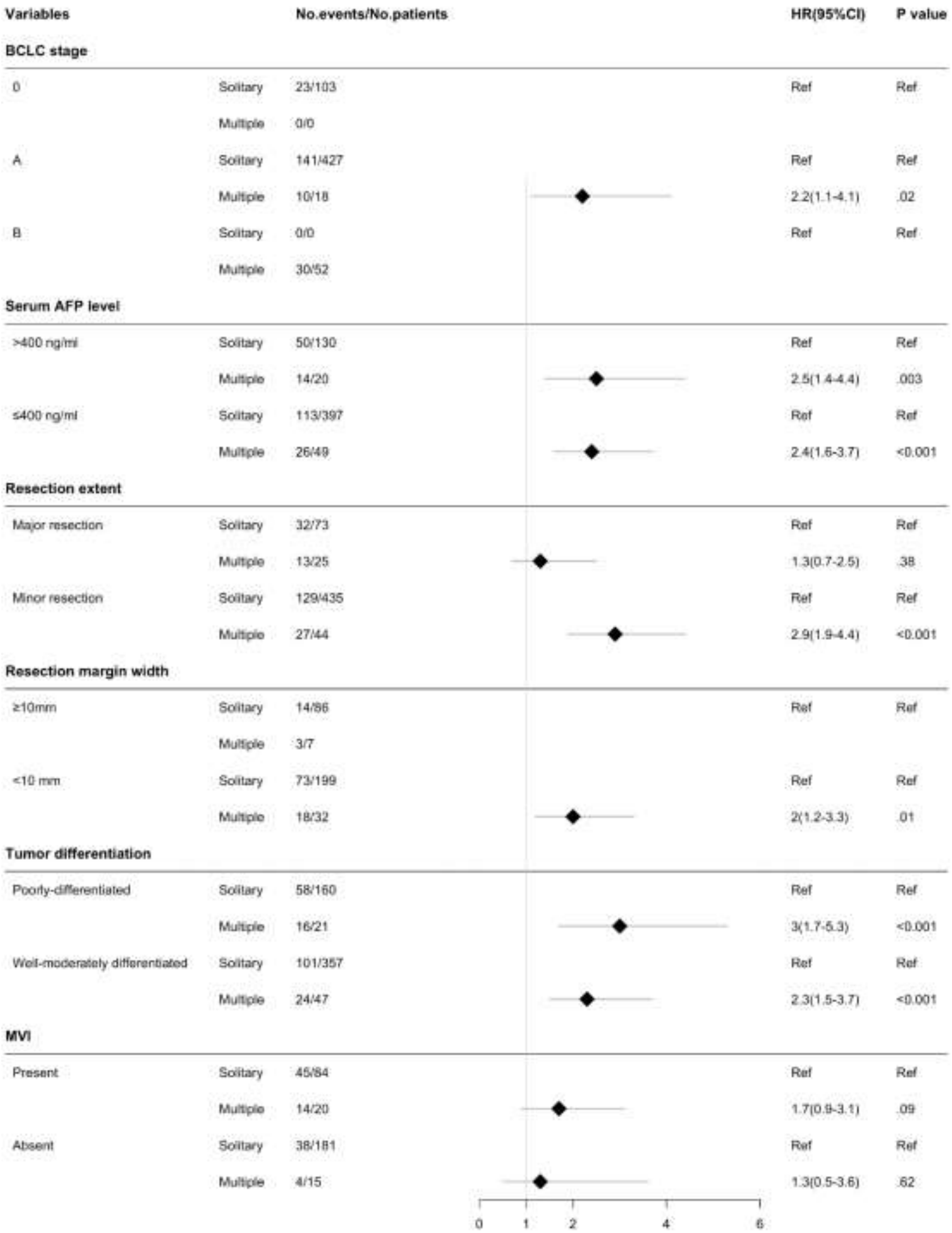

Supplemental Figure 4. Subgroup analyses for tumor number (solitary vs. multiple) in predicting early recurrence-free survival based on six clinical-pathological prognostic factors. BCLC, Barcelona Clinic Liver Cancer; AFP, α-fetoprotein; MVI, microvascular invasion.

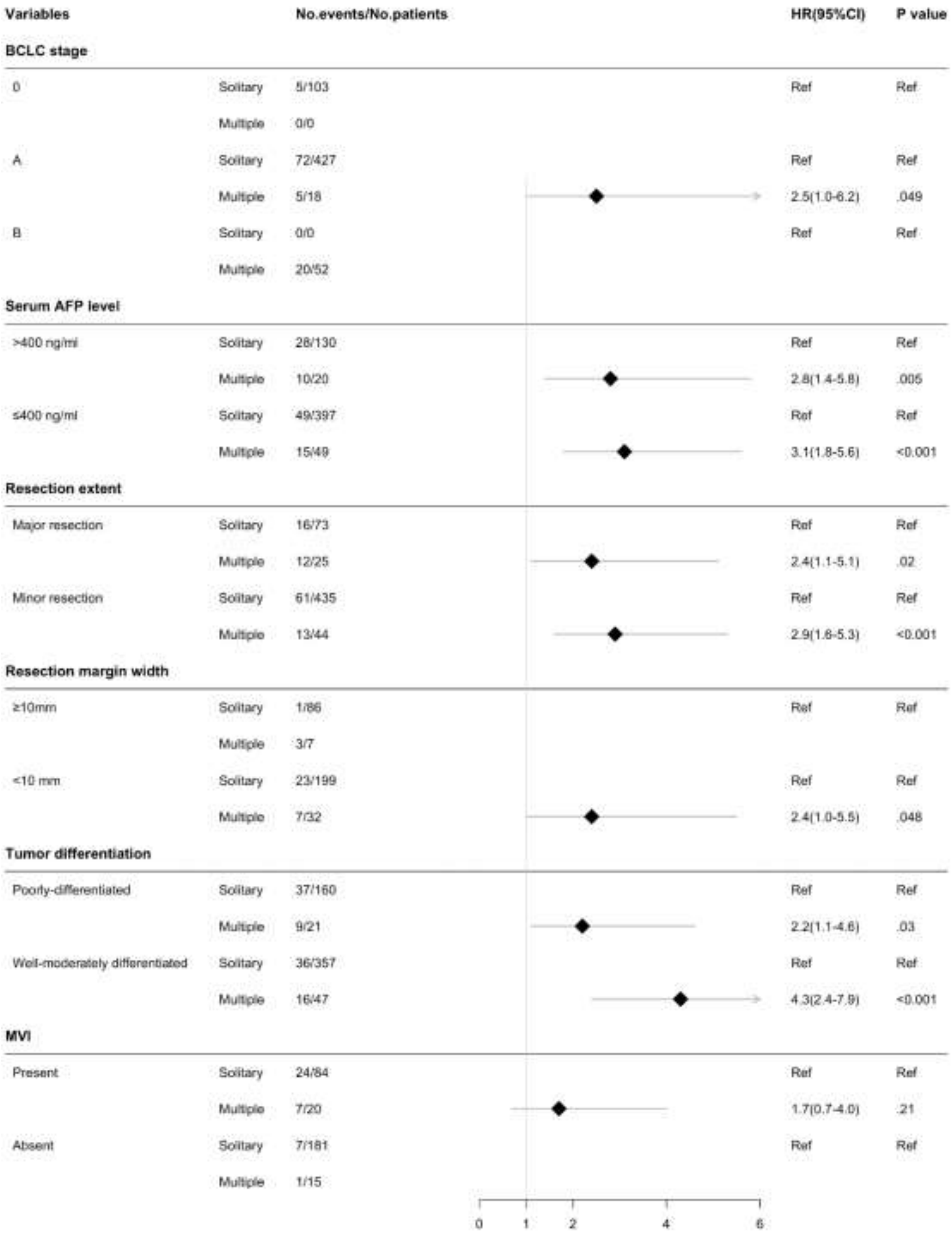

Supplemental Figure 5. Subgroup analyses for tumor number (solitary vs. multiple) in predicting overall survival based on six clinical-pathological prognostic factors.

BCLC, Barcelona Clinic Liver Cancer; AFP, α-fetoprotein; MVI, microvascular invasion.

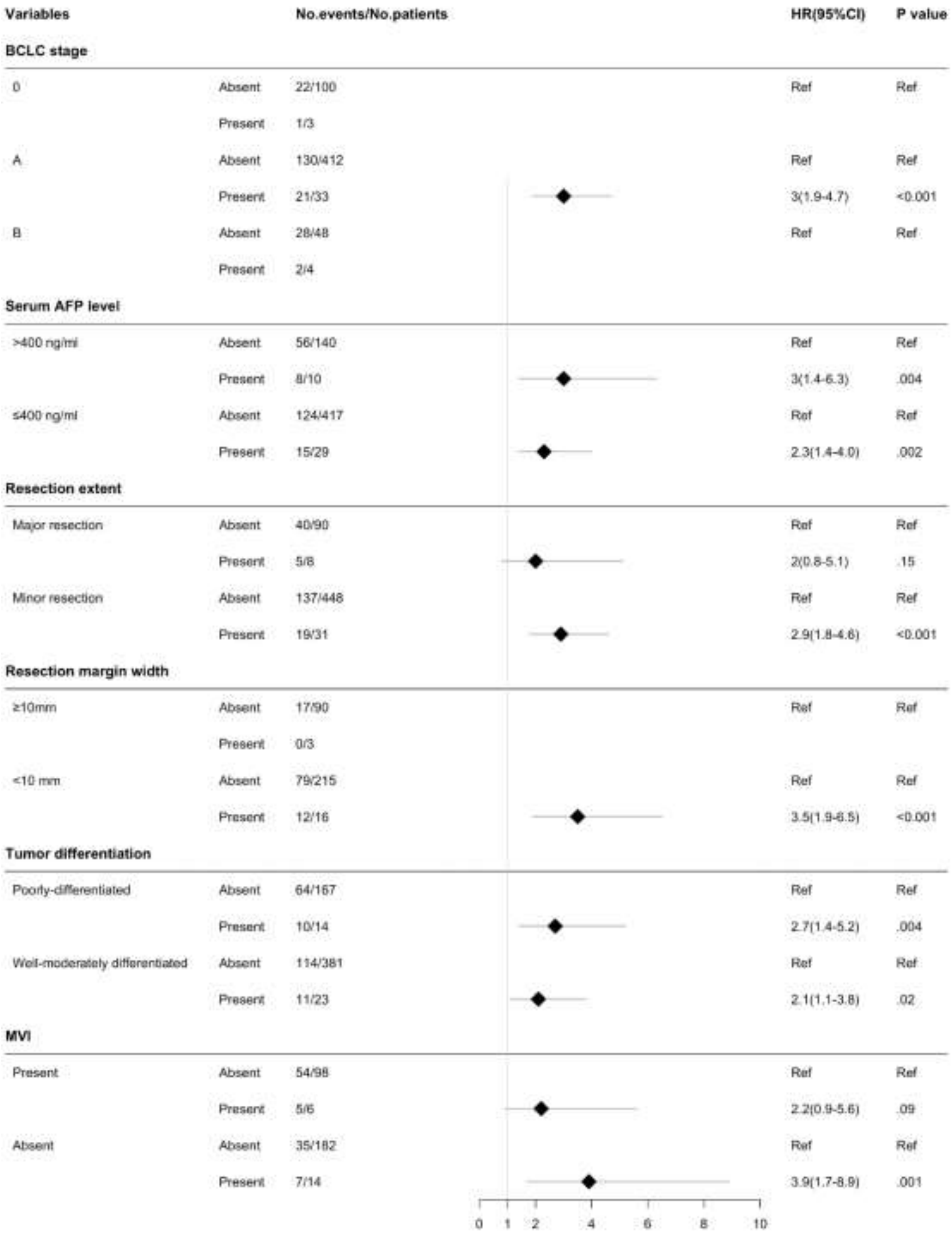

Supplemental Figure 6. Subgroup analyses for rim arterial phase hyperenhancement (present vs. absent) in predicting early recurrence-free survival based on six clinical-pathological prognostic factors.

BCLC, Barcelona Clinic Liver Cancer; AFP, α-fetoprotein; MVI, microvascular invasion.

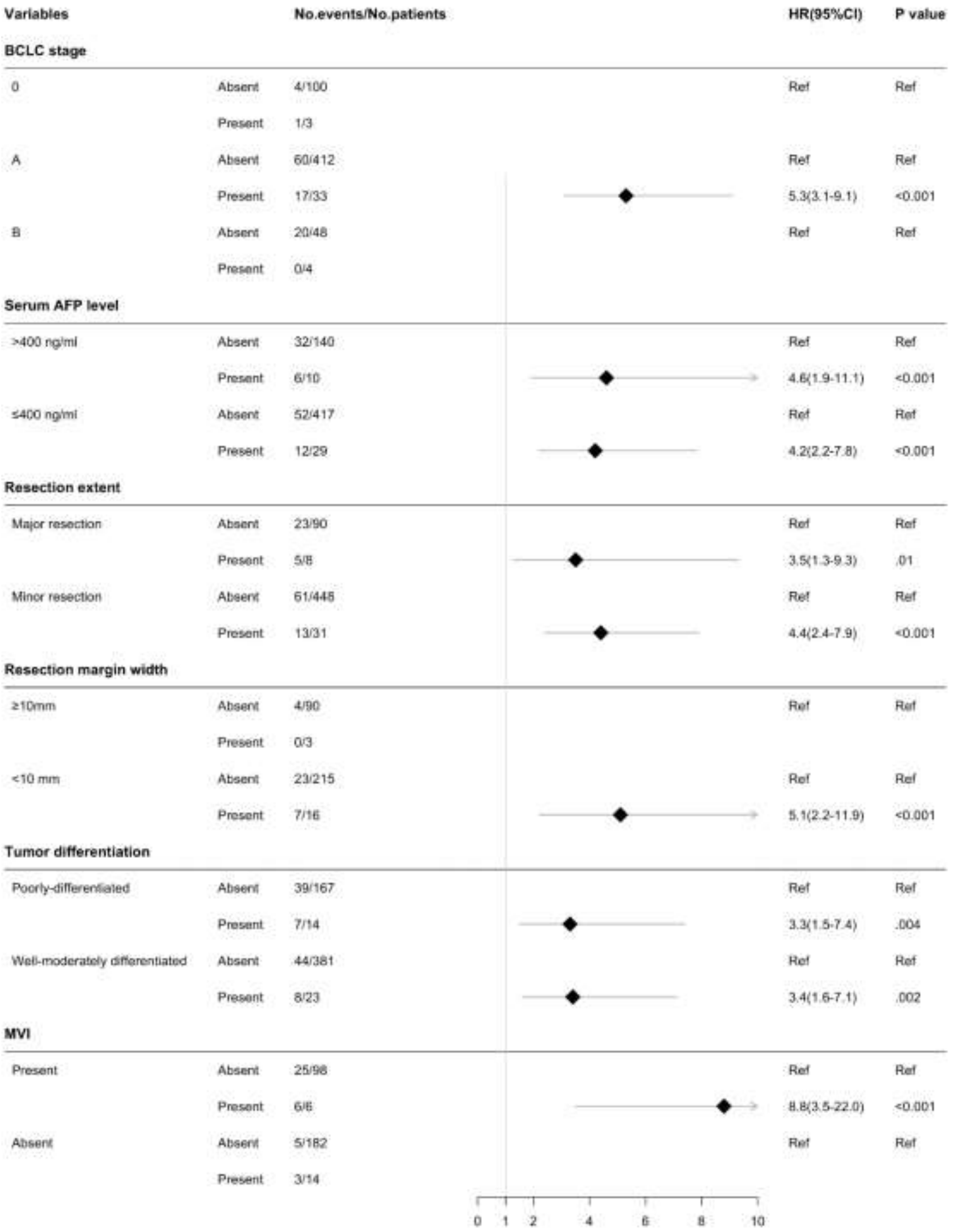

Supplemental Figure 7. Subgroup analyses for rim arterial phase hyperenhancement (present vs. absent) in predicting overall survival based on six clinical-pathological prognostic factors.

BCLC, Barcelona Clinic Liver Cancer; AFP, α-fetoprotein; MVI, microvascular invasion.

Variables		No.events/No.patients	HR(95%CI)	P value
**BCLC stage**				
0	Absent	17/87	Ref	Ref
	Present	6/16	1.9(0.7-4.7)	.19
A	Absent	122/383	Ref	Ref
	Present	29/62	1.7(1.1-2.6)	.010
B	Absent	18/37	Ref	Ref
	Present	12/15	2.4(1.1-5.0)	.02
**Serum AFP level**				
>400 ng/ml	Absent	46/128	Ref	Ref
	Present	18/22	3.1(1.8-5.3)	<0.001
≤400 ng/ml	Absent	110/376	Ref	Ref
	Present	29/70	1.6(1.1-2.4)	.03
**Resection extent**				
Major resection	Absent	36/86	Ref	Ref
	Present	9/12	2.5(1.2-5.2)	.01
Minor resection	Absent	119/400	Ref	Ref
	Present	37/79	1.8(1.2-2.6)	.002
**Resection margin width**				
≥10mm	Absent	16/84	Ref	Ref
	Present	1/9		
<10 mm	Absent	68/190	Ref	Ref
	Present	23/41	1.8(1.1-2.8)	.02
**Tumor differentiation**				
Poorly-differentiated	Absent	57/154	Ref	Ref
	Present	17/27	2.1(1.2-3.8)	.008
Well-moderately differentiated	Absent	95/339	Ref	Ref
	Present	30/65	1.9(1.3-2.9)	.002
**MVI**				
Present	Absent	46/83	Ref	Ref
	Present	13/21	1.2(0.6-2.2)	.56
Absent	Absent	31/163	Ref	Ref
	Present	11/33	2(1.0-3.9)	.05

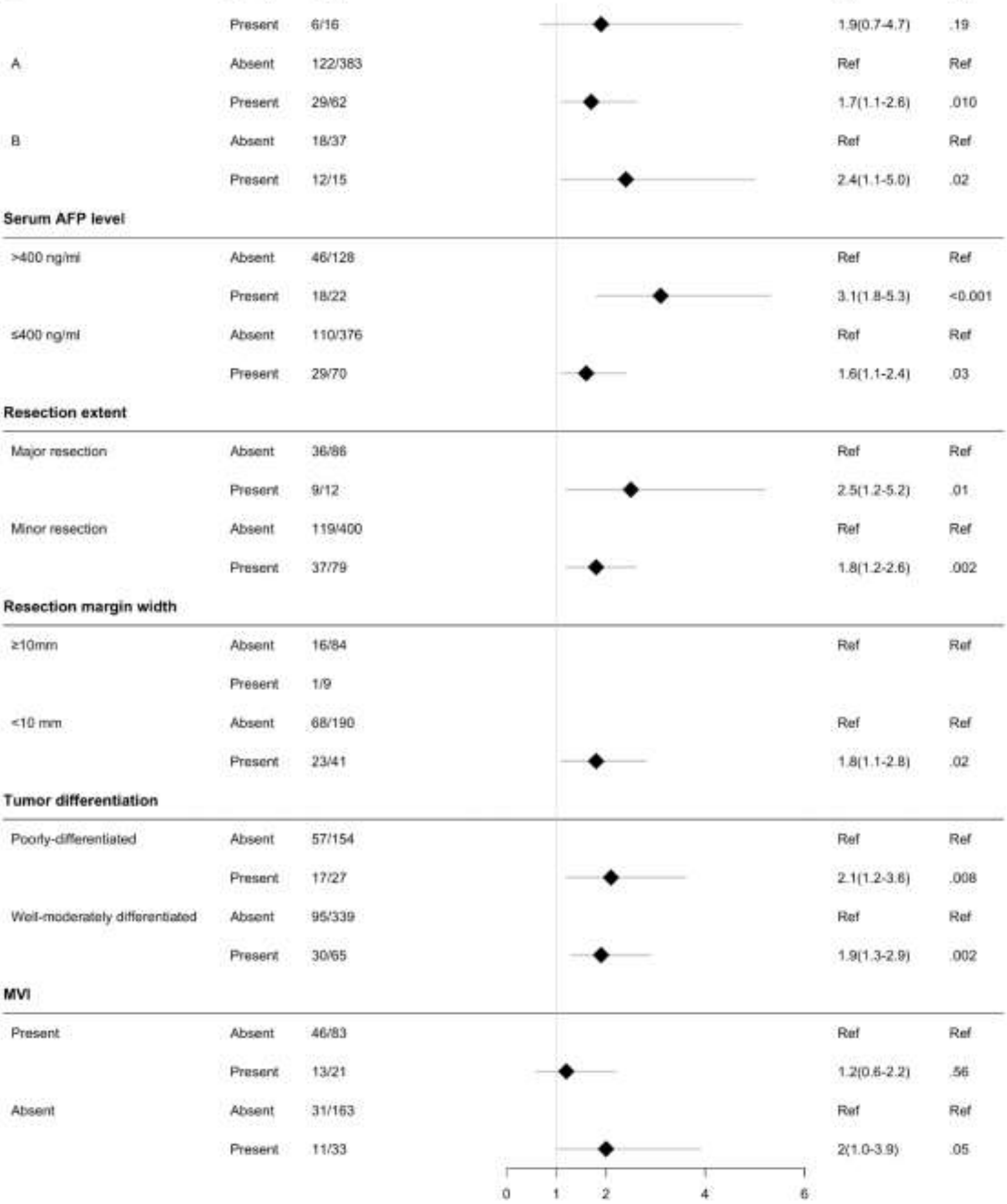

Supplemental Figure 8. Subgroup analyses for iron sparing in solid mass (present vs. absent) in predicting early recurrence-free survival based on six clinical-pathological prognostic factors.

BCLC, Barcelona Clinic Liver Cancer; AFP, α-fetoprotein; MVI, microvascular invasion.

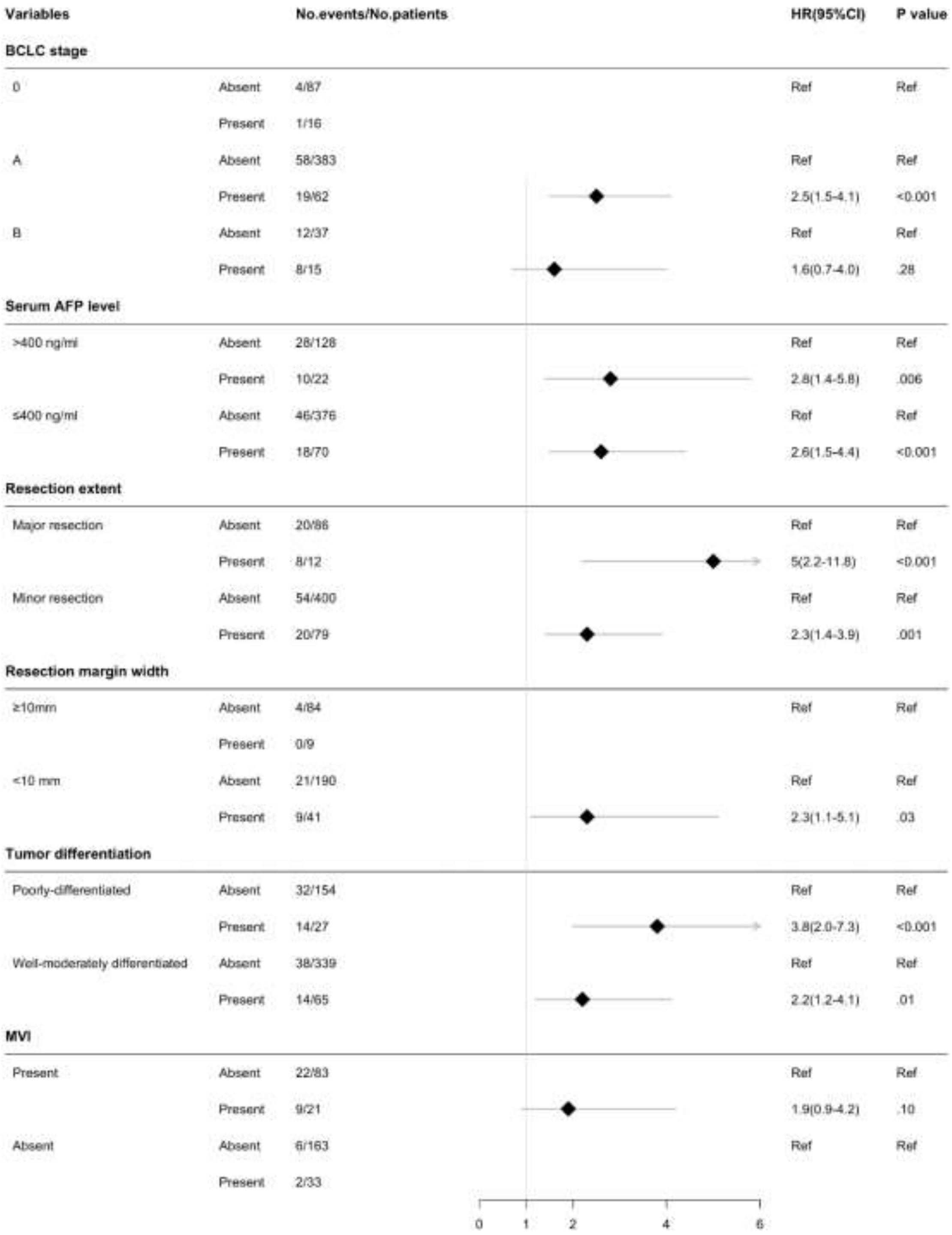

Supplemental Figure 9. Subgroup analyses for iron sparing in solid mass (present vs. absent) in predicting overall survival based on six clinical-pathological prognostic factors. BCLC, Barcelona Clinic Liver Cancer; AFP, α-fetoprotein; MVI, microvascular invasion.

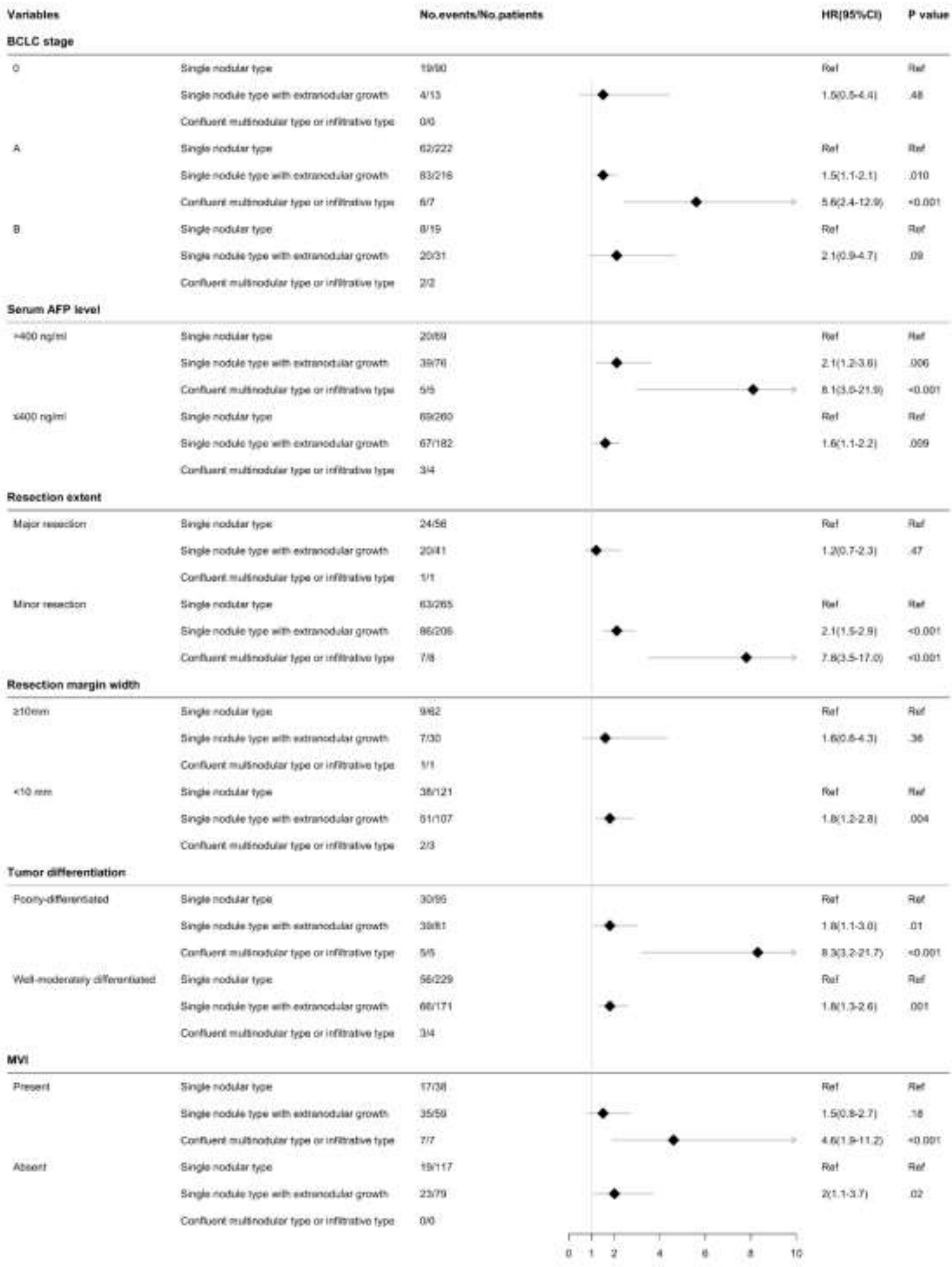

Supplemental Figure 10. Subgroup analyses for tumor growth subtype in predicting early recurrence-free survival based on six clinical-pathological prognostic factors.

BCLC, Barcelona Clinic Liver Cancer; AFP, α-fetoprotein; MVI, microvascular invasion.

Variables		No.events/No.patients	HR(95%CI)	P value
**BCLC stage**				
0	Single nodular type	4/90	Ref	Ref
	Single nodule type with extranodular growth	1/13		
	Confluent multinodular type or infiltrative type	0/0		
A	Single nodular type	26/222	Ref	Ref
	Single nodule type with extranodular growth	46/216	2(1.3-3.3)	.004
	Confluent multinodular type or infiltrative type	5/7	11(4.2-28.8)	<0.001
B	Single nodular type	5/18	Ref	Ref
	Single nodule type with extranodular growth	14/31	2.6(0.9-7.2)	.07
	Confluent multinodular type or infiltrative type	1/2		
**Serum AFP level**				
>400 ng/ml	Single nodular type	11/89	Ref	Ref
	Single nodule type with extranodular growth	23/76	2.2(1.1-4.4)	.04
	Confluent multinodular type or infiltrative type	4/5	9.4(2.9-30.0)	<0.001
≤400 ng/ml	Single nodular type	24/260	Ref	Ref
	Single nodule type with extranodular growth	38/182	2.5(1.5-4.1)	<0.001
	Confluent multinodular type or infiltrative type	2/4		
**Resection extent**				
Major resection	Single nodular type	13/56	Ref	Ref
	Single nodule type with extranodular growth	14/41	1.5(0.7-3.1)	.32
	Confluent multinodular type or infiltrative type	1/1		
Minor resection	Single nodular type	22/265	Ref	Ref
	Single nodule type with extranodular growth	47/206	3.1(1.9-5.1)	<0.001
	Confluent multinodular type or infiltrative type	5/8	13.5(5.1-36.0)	<0.001
**Resection margin width**				
≥10mm	Single nodular type	2/62	Ref	Ref
	Single nodule type with extranodular growth	2/30		
	Confluent multinodular type or infiltrative type	0/1		
<10 mm	Single nodular type	9/121	Ref	Ref
	Single nodule type with extranodular growth	19/107	3.1(1.4-6.9)	.005
	Confluent multinodular type or infiltrative type	2/3		
**Tumor differentiation**				
Poorly-differentiated	Single nodular type	15/95	Ref	Ref
	Single nodule type with extranodular growth	27/81	2.5(1.3-4.6)	.005
	Confluent multinodular type or infiltrative type	4/5	7.8(2.6-23.7)	<0.001
Well-moderately differentiated	Single nodular type	19/229	Ref	Ref
	Single nodule type with extranodular growth	31/171	2.4(1.3-4.2)	.003
	Confluent multinodular type or infiltrative type	2/4		
**MVI**				
Present	Single nodular type	6/38	Ref	Ref
	Single nodule type with extranodular growth	20/59	2.3(0.9-5.7)	.08
	Confluent multinodular type or infiltrative type	5/7	6(1.8-19.6)	.003
Absent	Single nodular type	3/117	Ref	Ref
	Single nodule type with extranodular growth	5/79	3(0.7-12.8)	.13
	Confluent multinodular type or infiltrative type	0/0		

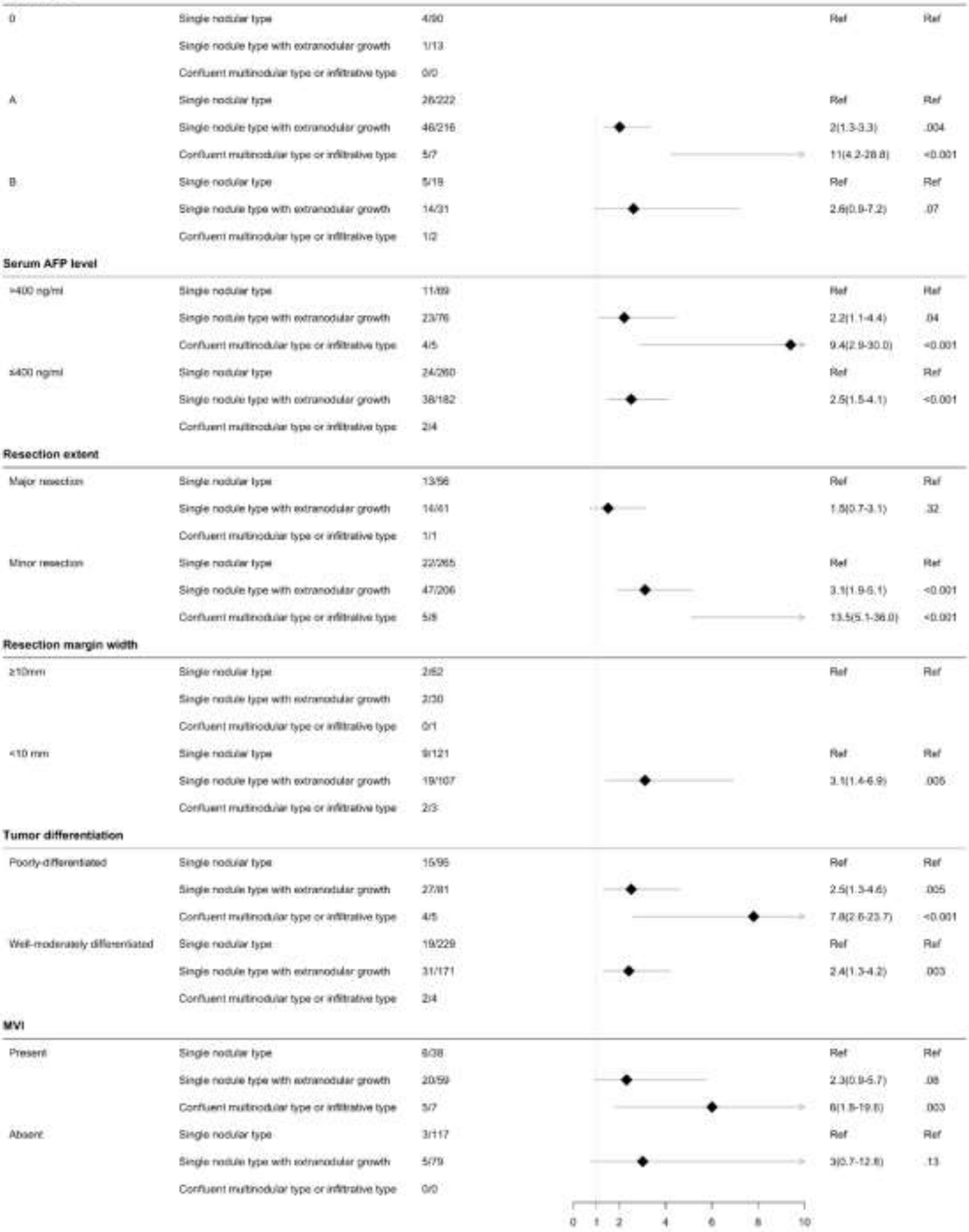

Supplemental Figure 11. Subgroup analyses for tumor growth subtype in predicting overall survival based on six clinical-pathological prognostic factors.

BCLC, Barcelona Clinic Liver Cancer; AFP, α-fetoprotein; MVI, microvascular invasion.

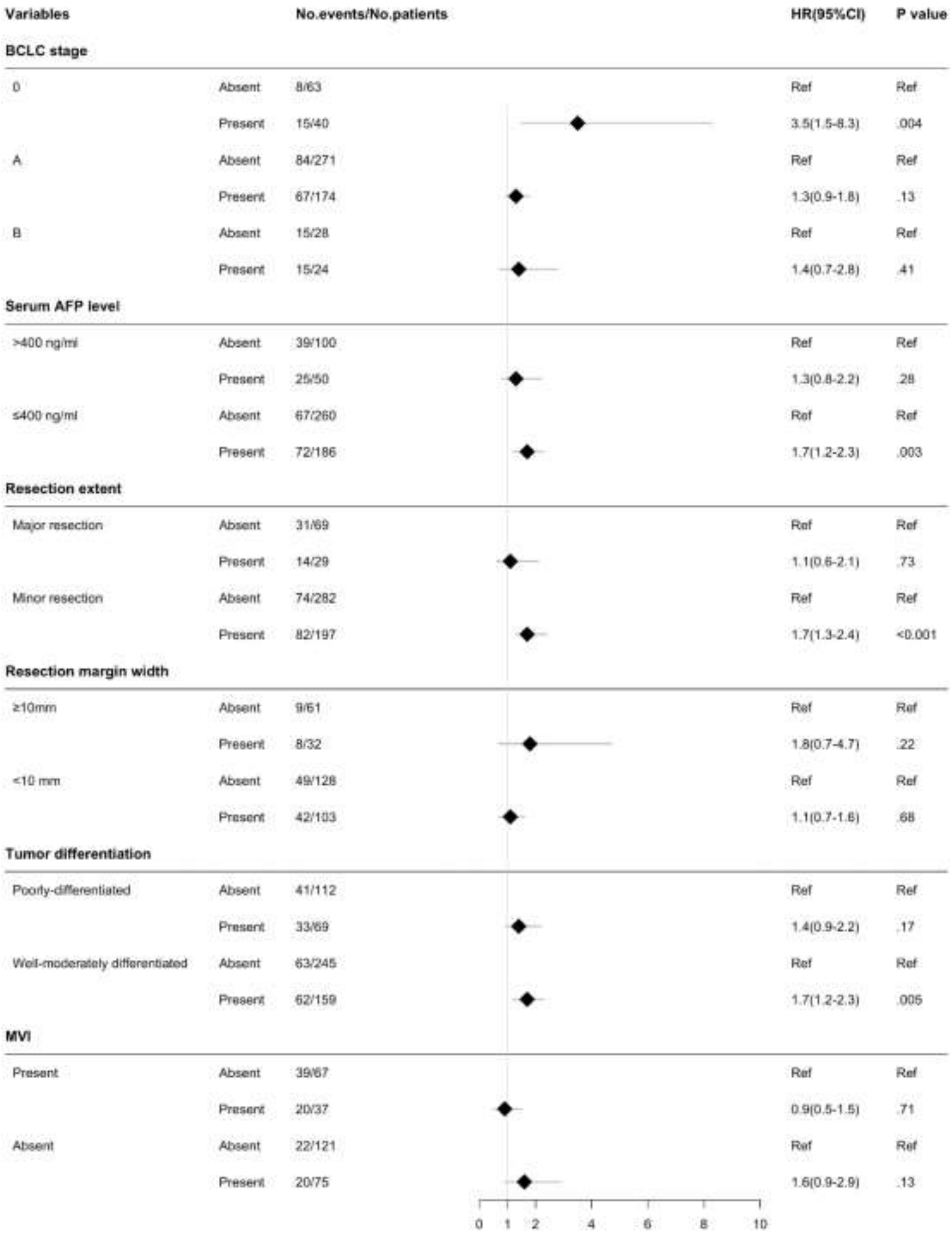

Supplemental Figure 12. Subgroup analyses for gastroesophageal varices (present vs. absent) in predicting early recurrence-free survival based on six clinical-pathological prognostic factors.

BCLC, Barcelona Clinic Liver Cancer; AFP, α-fetoprotein; MVI, microvascular invasion.

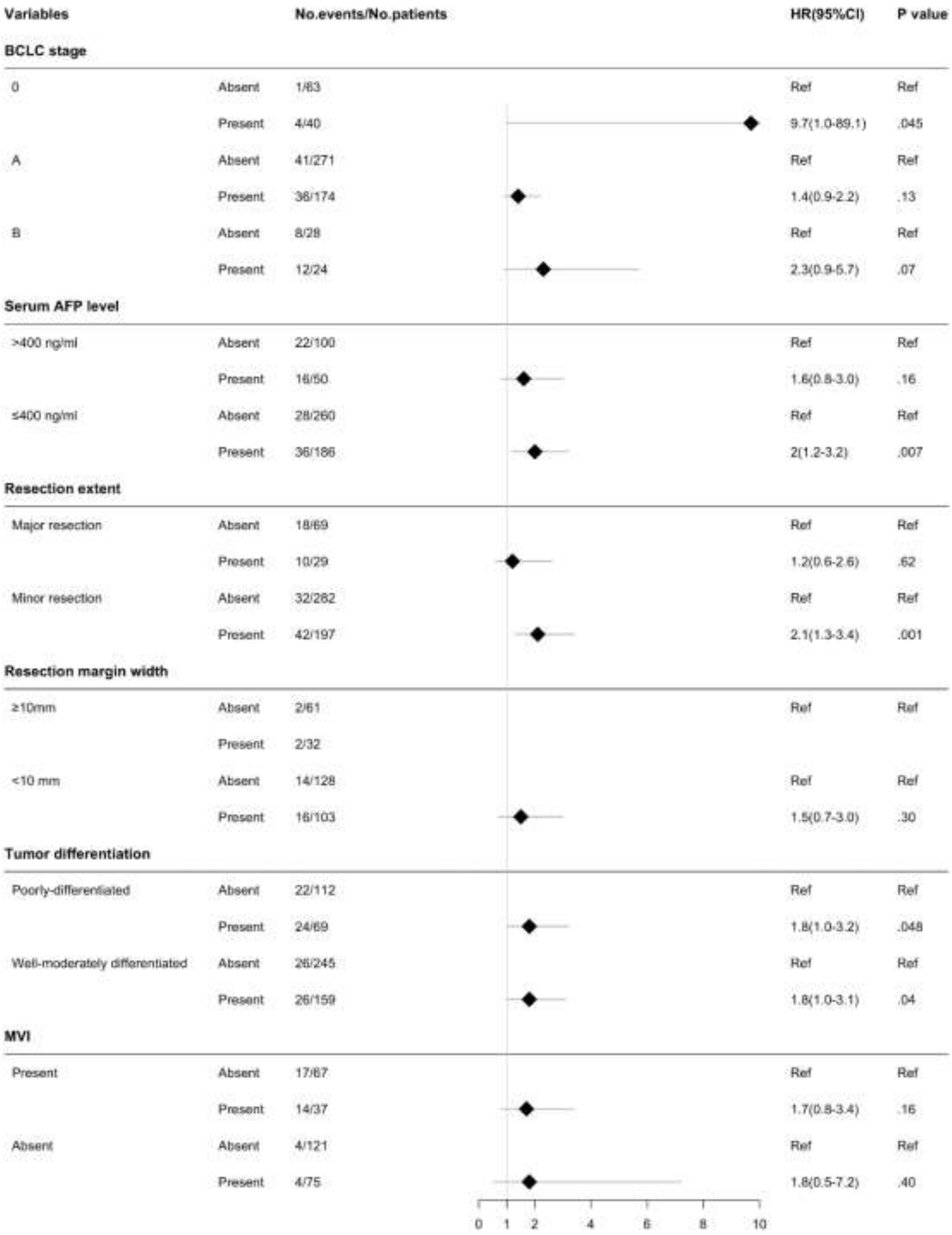

Supplemental Figure 13. Subgroup analyses for gastroesophageal varices (present vs. absent) in predicting overall survival based on six clinical-pathological prognostic factors. BCLC, Barcelona Clinic Liver Cancer; AFP, α-fetoprotein; MVI, microvascular invasion.

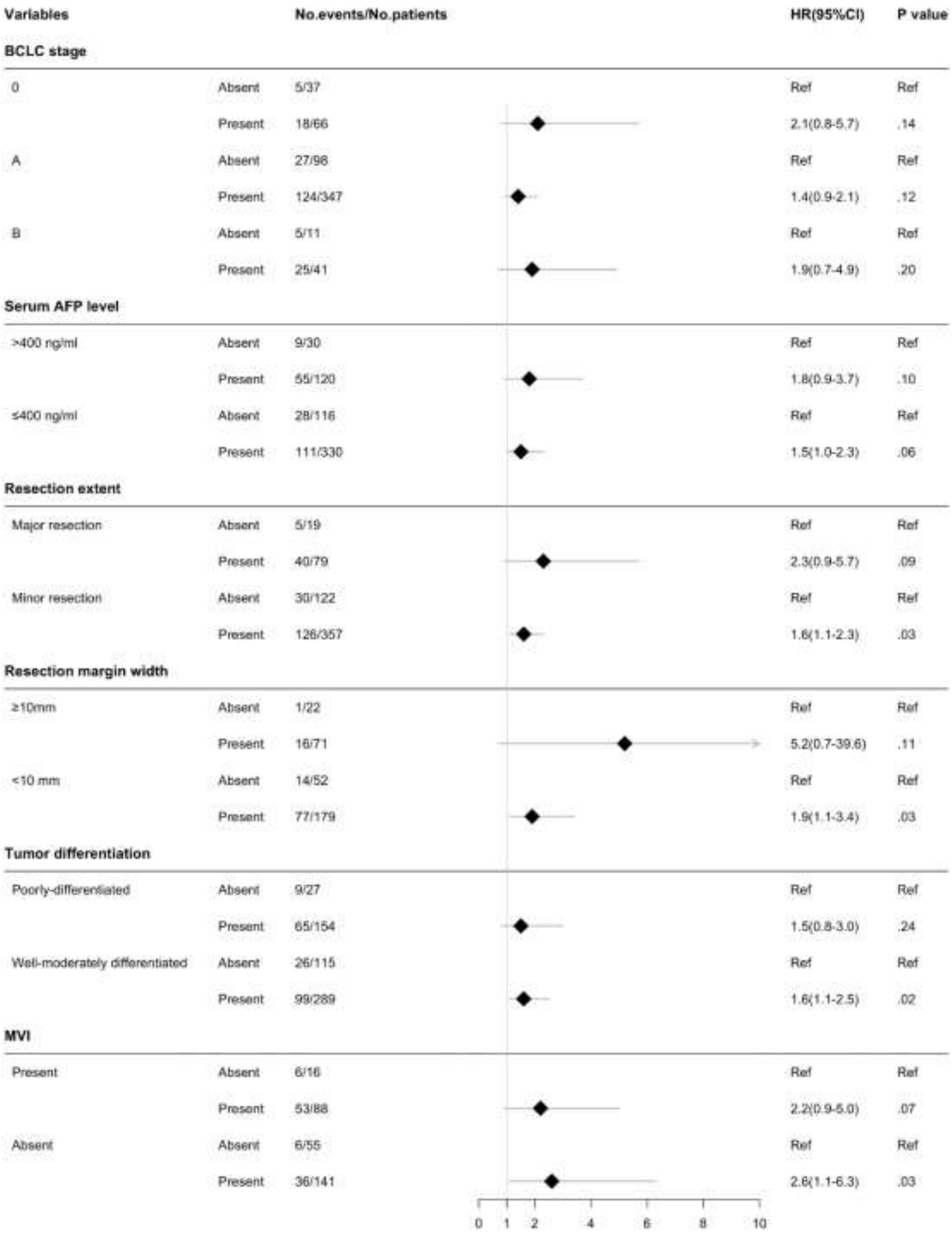

Supplemental Figure 14. Subgroup analyses for nonperipheral washout (present vs. absent) in predicting early recurrence-free survival based on six clinical-pathological prognostic factors.

BCLC, Barcelona Clinic Liver Cancer; AFP, α-fetoprotein; MVI, microvascular invasion.

Variables		No.events/No.patients	HR(95%CI)	P value
**BCLC stage**				
0	Absent	21/98	Ref	Ref
	Present	2/5		
A	Absent	132/410	Ref	Ref
	Present	19/35	1.9(1.2-3.1)	.007
B	Absent	25/47	Ref	Ref
	Present	5/5	4.1(1.6-10.9)	.004
**Serum AFP level**				
>400 ng/ml	Absent	55/139	Ref	Ref
	Present	9/11	3.6(1.8-7.2)	<0.001
≤400 ng/ml	Absent	122/412	Ref	Ref
	Present	17/34	1.9(1.1-3.1)	.01
**Resection extent**				
Major resection	Absent	38/87	Ref	Ref
	Present	7/11	1.6(0.7-3.6)	.24
Minor resection	Absent	137/445	Ref	Ref
	Present	19/34	2.2(1.4-3.6)	.001
**Resection margin width**				
≥10mm	Absent	14/86	Ref	Ref
	Present	3/7		
<10 mm	Absent	77/213	Ref	Ref
	Present	14/18	2.8(1.6-5.0)	<0.001
**Tumor differentiation**				
Poorly-differentiated	Absent	64/166	Ref	Ref
	Present	10/15	2.1(1.1-4.1)	.03
Well-moderately differentiated	Absent	110/375	Ref	Ref
	Present	15/29	2.1(1.2-3.7)	.006
**MVI**				
Present	Absent	48/92	Ref	Ref
	Present	11/12	2.8(1.4-5.4)	.002
Absent	Absent	37/183	Ref	Ref
	Present	5/13	2(0.8-5.1)	.15

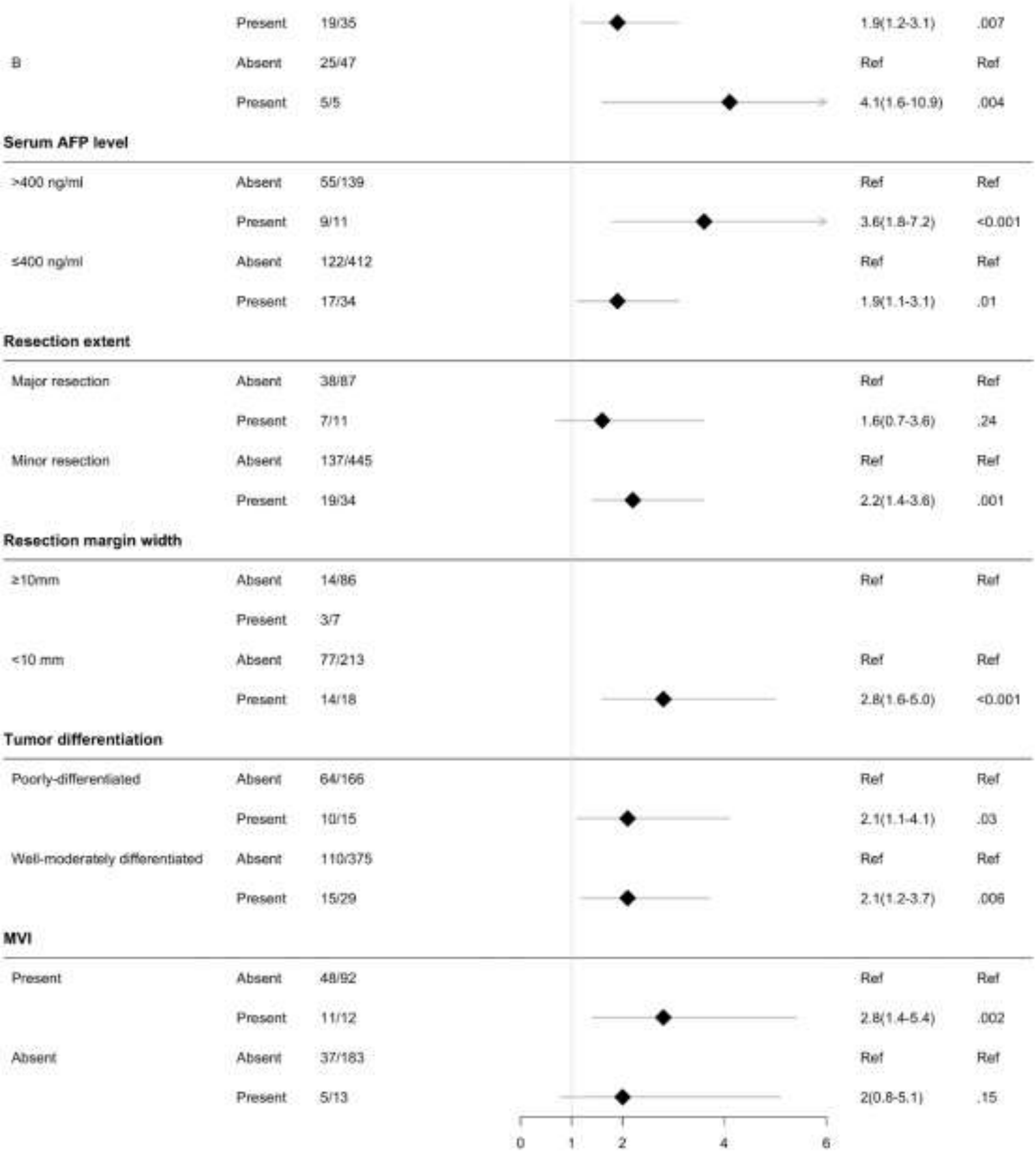

Supplemental Figure 15. Subgroup analyses for markedly low apparent diffusion coefficient value (present vs. absent) in predicting early recurrence-free survival based on six clinical-pathological prognostic factors.

BCLC, Barcelona Clinic Liver Cancer; AFP, α-fetoprotein; MVI, microvascular invasion.

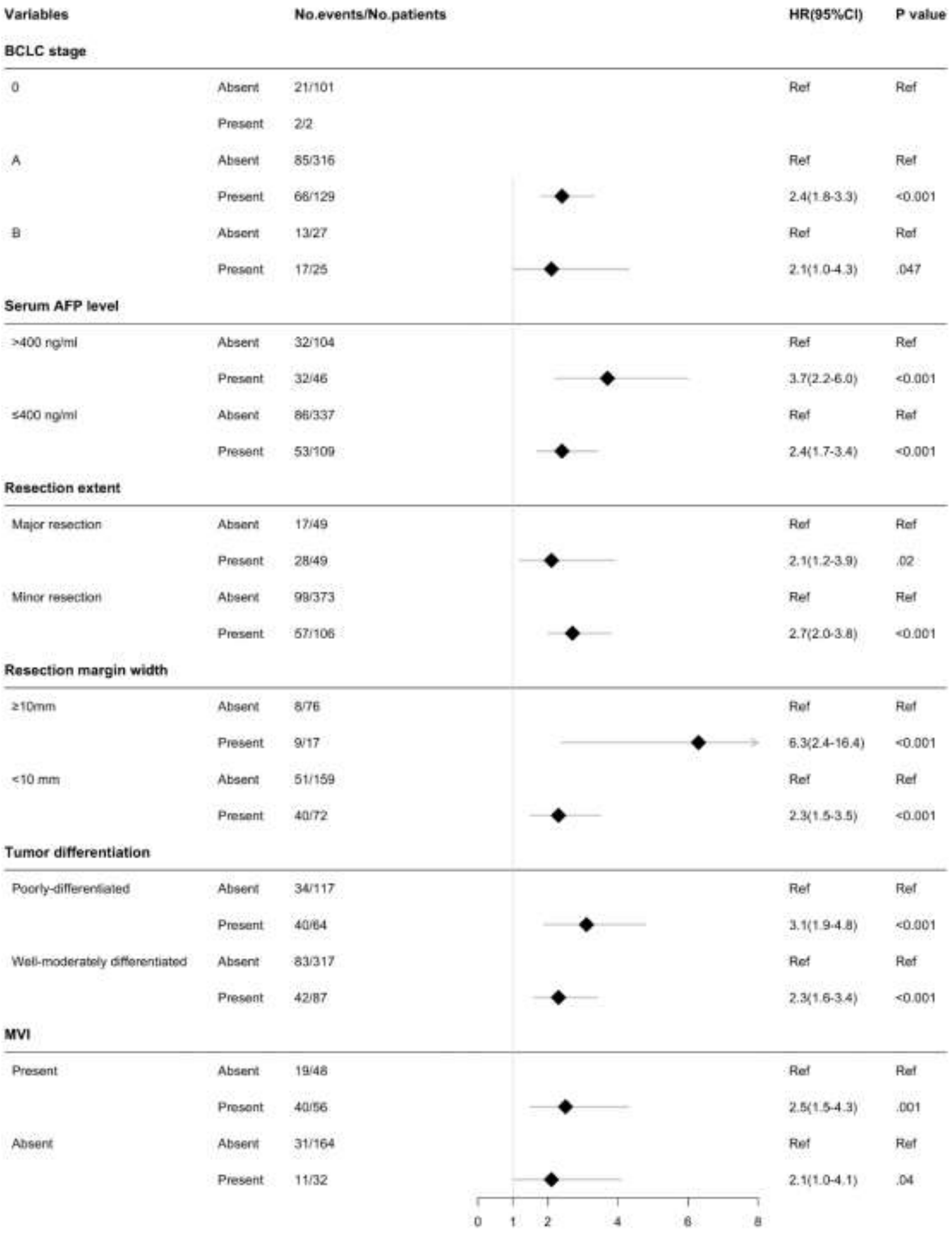

Supplemental Figure 16. Subgroup analyses for intratumoral artery (present vs. absent) in predicting early recurrence-free survival based on six clinical-pathological prognostic factors. BCLC, Barcelona Clinic Liver Cancer; AFP, α-fetoprotein; MVI, microvascular invasion.

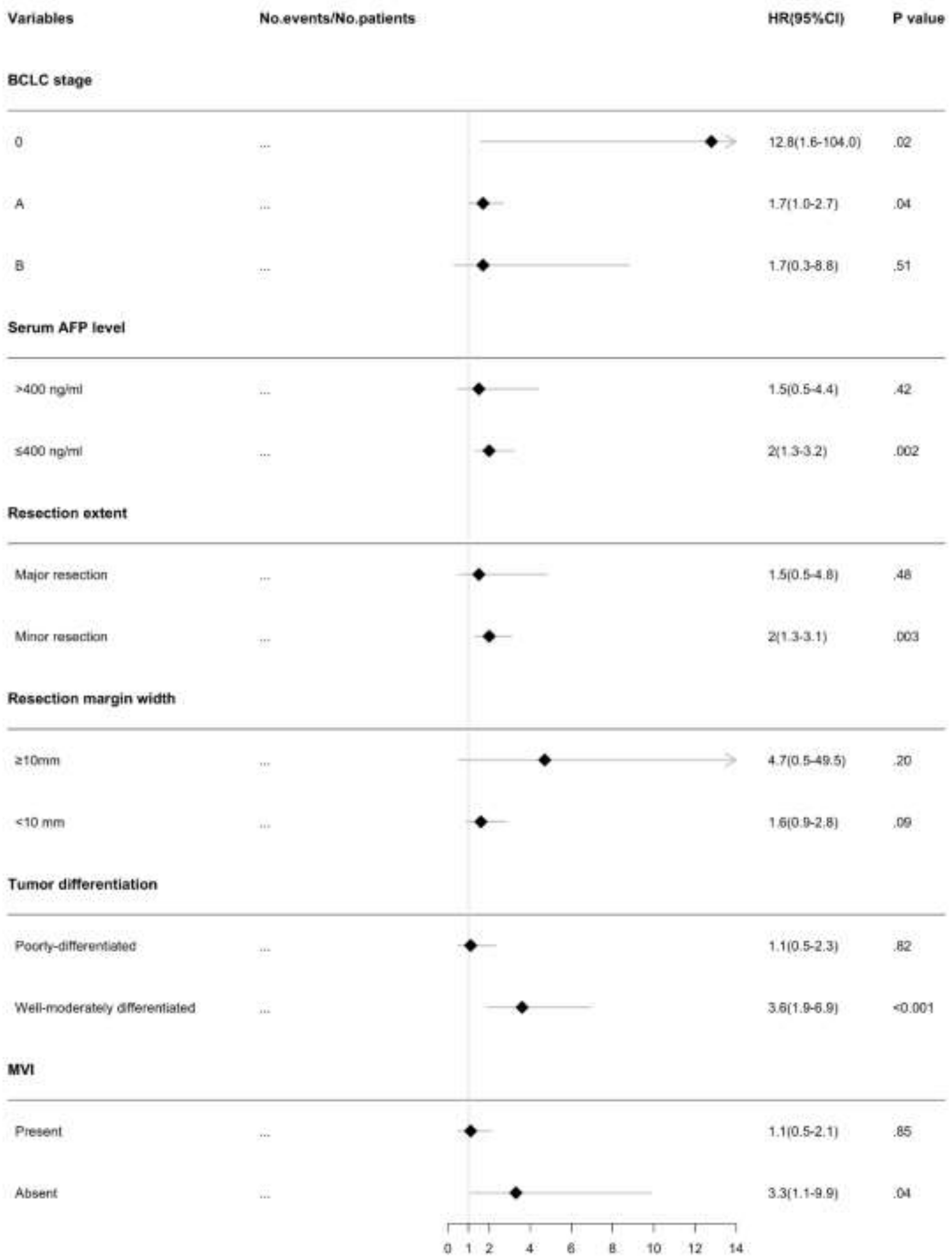

Supplemental Figure 17. Subgroup analyses for width of main portal vein (cm) in predicting early recurrence-free survival based on six clinical-pathological prognostic factors.

BCLC, Barcelona Clinic Liver Cancer; AFP, α-fetoprotein; MVI, microvascular invasion.

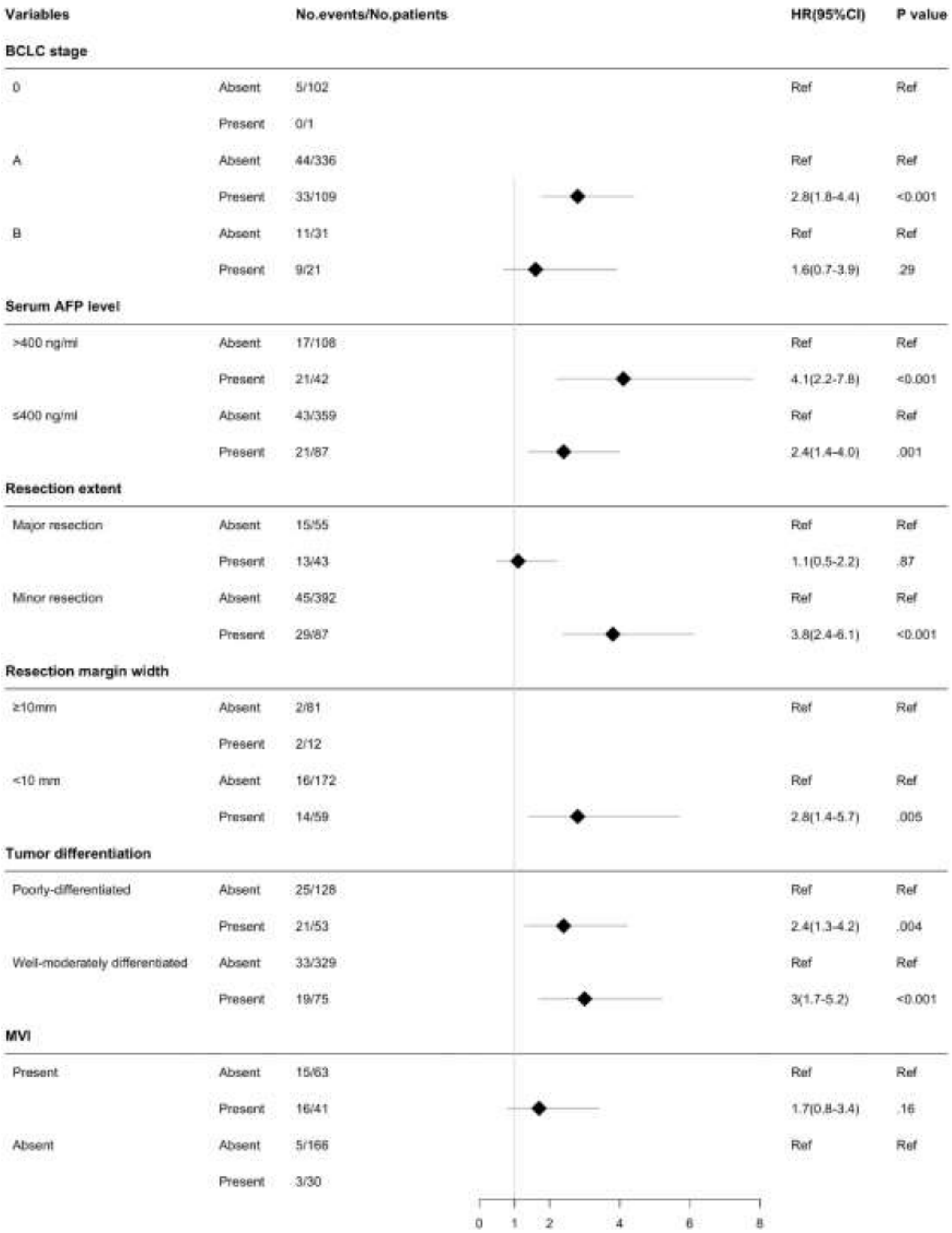

Supplemental Figure 18. Subgroup analyses for the VICT2 trait (present vs. absent) in predicting overall survival based on six clinical-pathological prognostic factors.

BCLC, Barcelona Clinic Liver Cancer; AFP, α-fetoprotein; MVI, microvascular invasion.
